# The functional response of human monocyte-derived macrophages to serum amyloid A and *Mycobacterium tuberculosis* infection

**DOI:** 10.3389/fimmu.2023.1238132

**Published:** 2023-09-15

**Authors:** Malwina Kawka, Renata Płocińska, Przemysław Płociński, Jakub Pawełczyk, Marcin Słomka, Justyna Gatkowska, Katarzyna Dzitko, Bożena Dziadek, Jarosław Dziadek

**Affiliations:** ^1^ Department of Molecular Microbiology, Faculty of Biology and Environmental Protection, University of Lodz, Lodz, Poland; ^2^ Institute of Medical Biology, Polish Academy of Sciences, Lodz, Poland; ^3^ Biobank Lab, Department of Oncobiology and Epigenetics, Faculty of Biology and Environmental Protection, University of Lodz, Lodz, Poland

**Keywords:** tuberculosis, human serum amyloid A, monocyte-derived macrophages, immunological response, host-pathogen transcriptomics

## Abstract

**Introduction:**

In the course of tuberculosis (TB), the level of major acute phase protein, namely serum amyloid A (hSAA-1), increases up to a hundredfold in the pleural fluids of infected individuals. Tubercle bacilli infecting the human host can be opsonized by hSAA-1, which affects bacterial entry into human macrophages and their intracellular multiplication.

**Methods:**

We applied global RNA sequencing to evaluate the functional response of human monocyte-derived macrophages (MDMs), isolated from healthy blood donors, under elevated hSAA-1 conditions and during infection with nonopsonized and hSAA-1-opsonized *Mycobacterium tuberculosis* (*Mtb*). In the same infection model, we also examined the functional response of mycobacteria to the intracellular environment of macrophages in the presence and absence of hSAA-1. The RNASeq analysis was validated using qPCR. The functional response of MDMs to hSAA-1 and/or tubercle bacilli was also evaluated for selected cytokines at the protein level by applying the Milliplex system.

**Findings:**

Transcriptomes of MDMs cultured in the presence of hSAA-1 or infected with *Mtb* showed a high degree of similarity for both upregulated and downregulated genes involved mainly in processes related to cell division and immune response, respectively. Among the most induced genes, across both hSAA-1 and *Mtb* infection conditions, CXCL8, CCL15, CCL5, IL-1β, and receptors for IL-7 and IL-2 were identified. We also observed the same pattern of upregulated pro-inflammatory cytokines (TNFα, IL-6, IL-12, IL-18, IL-23, and IL-1) and downregulated anti-inflammatory cytokines (IL-10, TGFβ, and antimicrobial peptide cathelicidin) in the hSAA-1 treated-MDMs or the phagocytes infected with tubercle bacilli. At this early stage of infection, *Mtb* genes affected by the inside microenvironment of MDMs are strictly involved in iron scavenging, adaptation to hypoxia, low pH, and increasing levels of CO_2_. The genes for the synthesis and transport of virulence lipids, but not cholesterol/fatty acid degradation, were also upregulated.

**Conclusion:**

Elevated serum hSAA-1 levels in tuberculosis enhance the response of host phagocytes to infection, including macrophages that have not yet been in contact with mycobacteria. SAA induces antigen processing and presentation processes by professional phagocytes reversing the inhibition caused by *Mtb* infection.

## 1 Introduction

*Mycobacterium tuberculosis* (*Mtb*) is a causative agent of tuberculosis (TB), an infectious disease that affects millions of people worldwide. TB remains a major global health problem, with an estimated 10.6 million cases globally and 1.6 million deaths worldwide in 2021, according to the World Health Organization (WHO) Global Tuberculosis Report 2022 (1). *Mtb* is a slow-growing bacterium with a complex cell wall that makes it resistant to many antibiotics and allows it to evade the host’s immune system (2). The transmission of TB occurs through inhalation of respiratory droplets containing bacteria, which are excreted from an infected person during coughing, sneezing or talking. The bacteria can then infect the lungs and other parts of the body of a newly infected individual, leading to the development of TB (2, 3). The pathogenesis of TB is complex and involves a network of interactions between the bacterium and the host’s immune response. Several factors contribute to the development of active TB, including host genetics, environmental factors, and the virulence of the infecting strain. The disease can cause distress to multiple organs, but primarily affects the lungs, leading to symptoms such as cough, fever, and weight loss (4, 5). The immune response to *Mtb* involves a complex interplay between the innate and adaptive immune systems, which work together to control the infection. The initial immune response to *Mtb* is mediated by innate immune cells, including macrophages and dendritic cells, capable of engulfing and phagocytosing the bacteria. Once inside the phagosome, *Mtb* evades eradication by manipulating the host immune response, preventing acidification of the phagosome and avoiding exposure to lysosomal enzymes. To counteract these strategies, innate immune cells secrete cytokines and chemokines that recruit other immune cells to the site of infection, including neutrophils, natural killer cells, and T cells. This results in the formation of granulomas, which are dense, granule-like aggregates of immune cells that surround the infected macrophages and sequester the bacteria. In granulomas *Mtb* can enter a dormant state where it can persist for years without causing disease. Reactivation of the bacterial infection can occur if the immune system becomes compromised, as in the case of HIV coinfection or immunosuppressive therapy, leading to the development of TB (6, 7). The adaptive immune response to *Mtb* is primarily mediated by CD4^+^ T-cells, which recognize tubercle bacilli antigens presented by antigen-presenting cells (APCs) e.g. Ag85B (8), a wide range of peptides from proteins of secretion systems ESX-1, ESX-3, ESX-5 and also membrane-bound protease FtsH and putative conserved ATPase (9, 10). As for the group of *Mtb* non-protein components presented by APC, it has been shown to belong to it monoglycosolated mycolic acids, phospatydilinositols (e.g. LAM, PIM2, and PIM6), and diacylated sulfoglycolipids (11). Once activated, CD4^+^ T-cells differentiate into T helper 1 (Th1) cells, which secrete cytokines such as interferon-gamma (IFN-γ) and tumor necrosis factor-alpha (TNF-α) that activate macrophages and enhance their bactericidal activity (12, 13). In addition to Th1 cells, other CD4^+^ T cell subsets, such as Th17 and regulatory T-cells, also play a role in the immune response to *Mtb*. Th17 cells secrete cytokines including IL-17 that recruit neutrophils and enhance their ability to kill *Mtb*, while regulatory T cells help to dampen the inflammatory response and prevent tissue damage. However, the precise role of Th17 cells during *Mtb* infection is not fully clear due to their potential contribution to tuberculosis pathology and progression (14, 15).

Serum amyloid A (SAA), together with C-reactive protein, is classified as a positive, major acute-phase protein that is produced mainly by liver hepatocytes, and is present in low concentrations (1-2 µg/mL) in the blood of healthy individuals. However, during inflammation or infection, the concentration of SAA can increase dramatically within the first 4 hours, reaching values up to 1000-fold higher than baseline levels. Among the four isoforms of human SAA, namely SAA1, SAA2, SAA3 and SAA4, only two of them, SAA1 and SAA2, are considered acute phase proteins (A-SAA) (16). During the inflammatory response, mediators of A-SAA synthesis are endogenous and exogenous factors including IL-6, IL-1, TNF-α, bacterial endotoxin, and glucocorticoids (17–19). The high evolutionary conserved nature among vertebrates, low physiological serum concentration, and lack of documented deficiencies related to A-SAA indicate a significant biological role of this acute-phase protein. A-SAA is characterized by pleiotropic functional activity necessary to maintain homeostasis, which is associated not only with its high immunomodulatory potential but also with the involvement of A-SAA in tissue repair processes. Engagement of A-SAA in tissue regeneration and repair relay on its role in the mobilization of cholesterol, and functioning as an angiogenic and retinol-binding protein (16, 20). The immunomodulatory functions of A-SAA depend on its proinflammatory and anti-inflammatory properties related to, inter alia, stimulation of the synthesis of many cytokines (e.g., TNF-α, IL-1β, IL-6, IL-23, GM-CSF, IL-10) and chemokines (e.g., CXCL8, CCL2), and an ability to activate the NLRP3 inflammasome of macrophages (17, 21). As a part of the innate immune system A-SAA plays an important role in the response to various infectious agents. The ability of A-SAA to opsonize pathogenic microorganisms allows the classification of this protein as one of the circulating pattern recognition receptors (PRRs) recognizing pathogen-associated molecular patterns (PAMPs). The specific binding interactions with A-SAA were described for gram-negative bacteria, including *Escherichia coli*, *Salmonella enterica*, *Shigella flexneri*, *Pseudomonas aeruginosa*, *Vibrio cholerae*, *Klebsiella pneumoniae* and *Serratia marcescens*. Furthermore, bacterial proteins, namely OmpA (*E. coli*) and its homolog OprF (*P. aeruginosa*) were identified as A-SAA ligands (22). Further study revealed that opsonization of gram-negative bacteria with A-SAA at physiological concentrations of this protein promotes their phagocytosis by neutrophils and macrophages and stimulates inflammatory mechanisms of professional phagocytes relying on increased synthesis of IL-10 and TNF-α (23).

More recently we described the specific interaction of human A-SAA (SAA1) with *Mtb* and identified 5 mycobacterial membrane proteins, namely AtpA (Rv1308), ABC (Rv2477c), EspB (Rv3881c), TB18.6 (Rv2140c) and ThiC (Rv0423c) as the pathogen effectors responsible for this interaction. The opsonization of tubercle bacilli with SAA1 favored bacterial entry into human monocyte-derived macrophages (MDMs), accompanied by a substantial increase in the load of intracellularly multiplying and surviving bacteria (24).

Here, using global transcriptomics analyses, we evaluated the functional response of human MDMs, isolated from the blood of healthy donors under elevated SAA-1 conditions and during infection with nonopsonized and SAA1-opsonized tubercle bacilli. Furthermore, we examined the functional response of mycobacteria to the intracellular environment of MDMs in the presence and absence of SAA1.

## 2 Materials and methods

### 2.1 Preparation of human MDMs and infection with *Mtb* and stimulation with human SAA1

Human monocytes were isolated from commercially available (Regional Blood Donation Station, Lodz, Poland) and freshly prepared buffy coats from anonymous healthy human blood donors (24, 25). Briefly, differentiation of MDMs was performed within 6 days of culturing 2.5 × 10^6^ blood monocytes in 2.5 mL of IMDM medium (Cytogen GmbH, Greven, Germany) containing 10 ng/mL macrophage colony-stimulating factor (M-CSF), (Thermo Fisher Scientific, Waltham, MA, USA) in 6-well tissue plates (Corning Incorporated, Corning, NY, USA) at 37°C in a humidified atmosphere of 10% CO_2_/90% air. Next, the cells were thoroughly washed three times to remove any nonadherent cells and antibacterial antibiotics and left to rest overnight.

We sought to determine the functional responses of MDMs to serum amyloid A and SAA-opsonized and nonopsonized *Mtb* at the transcriptome and cytokine levels as well as the transcriptional response of intracellularly located nonopsonized and opsonized tubercle bacilli. Live *Mtb* cells were subjected to initial interactions with human SAA1 (ProSpec-Tany TechnoGene Ltd., Ness-Ziona, Israel) at a final concentration of 15 µg/mL in IMDM containing 0.1% BSA (Sigma, St. Louis, MO, United States) and 3 mM CaCl_2_ (Sigma Aldrich) for 90 min at 37^○^C (warm water bath) with gentle shaking every 30 min. The MDMs were infected with tubercle bacilli, as described by Kawka et al. (24), or were stimulated with human SAA1. Briefly, prior to infection, the culture medium was replaced with 2 mL human serum-free medium supplemented with 0.2% BSA, employing three washes to remove the excess SAA1 present in human serum (SAA1 concentrations in human sera were assessed by an SAA human ELISA Kit, Hycult^®^Biotech, Wayne, USA). Then the separate MDM cultures were infected with nonopsonized and SAA1-opsonized *Mtb* at an MOI of 1:20 or were treated with human SAA1 at a final concentration of 15 µg/mL. After 2 h of incubation of *Mtb*-infected MDMs, at 37^○^C in a humidified atmosphere of 10% CO_2_/90% air, the extracellularly located tubercle bacilli were removed by extensive washing using IMDM medium. After 24 h of incubation supernatants were collected from the experimental and control cultures to determine the concentrations of selected cytokines. MDMs incubated with the medium alone and intracellularly located nonopsonized *Mtb* served as controls. To analyze the transcriptional response of MDMs and the pathogen, human cells were lysed with 2 mL of cold RLT Buffer (QIAGEN, Hilden, Germany) on ice for 5 min to isolate bacterial and human RNA.

Macrophages from four independent healthy blood donors were used to perform the experiments.

### 2.2 RNA extraction and RNA-Seq library construction

Following the infection cycle, macrophages were lysed in RNA later reagent (Invitrogen™, Walthman, MA, USA) following the manufacturer’s protocol and were then centrifuged at 8,000 rpm for 15 minutes at 4°C to collect cell debris and mycobacteria. The supernatant was mixed with 3 volumes of TRIzol LS Reagent (Thermo Fisher Scientific, Waltham, MA, USA) and macrophage RNA was isolated using the Direct-zol™ RNA MiniPrep Plus reagent kit (Zymo Research, Irvine, CA, USA) according to the manufacturer’s instructions. First, TRIzol cell lysate was mixed with an equal volume of 95% ethanol, transferred onto a Zymo-Spin IIC column and centrifuged. The column was washed with Direct-zol RNA PreWash, followed by RNA Wash Buffer and RNA was eluted with DNase/RNase Free Water. Total RNA from bacterial strains was isolated using TRIzol LS reagent as described previously (24, 26). Briefly, cells were disrupted twice using the MP disruptor system with the Quick prep adapter (MP Biomedicals, Irvine, CA, USA) and 0.1 mm silica spheres. DNA contamination of the RNA samples was removed using a TURBO DNA-*free*™ Kit (Thermo Fisher Scientific, Waltham, MA, USA) according to the manufacturer’s protocol. The integrity and quantity of RNA were examined using an Agilent 2100 BioAnalyzer fitted with an Agilent RNA 6000 Nano Kit, following the manufacturer’s instructions (Agilent Technologies, Santa Clara, CA, USA).

The total RNA sequencing libraries were prepared as described previously in Plocinski et al., 2019 with minor modifications. Briefly, 2 µg of AMPure XP (Becton Dickinson, Burlington, NC, USA) bead-purified RNA was treated with a Ribo-off rRNA Depletion Kit (Human/Mouse/Rat, Vazyme, Nanjing, Jiangsu, China) to deplete rRNA (including cytoplasmic 28S, 18S, 5S rRNA, and mitochondrial 12S, 5.8S rRNA) from human total RNA (Illumina, San Diego, CA, USA) following the protocol accompanying the kit. The Ribo-Zero rRNA Removal Kit (Illumina, San Diego, CA, USA) was applied in the case of bacterial RNA. The sequencing libraries were prepared following the manufacturer’s instructions for the KAPA Stranded RNA-Seq Kit, (KAPA Biosystems, Roche, Basel, Switzerland). The quantity and quality of libraries were inspected on an Agilent 2100 BioAnalyzer fitted with a DNA 1000 chip. The obtained cDNA libraries were sequenced using a NextSeq 500 System (Illumina and a NextSeq 500/550 Mid Output v2 Sequencing Kit (150 cycles), (Illumina, San Diego, CA, USA), thus guaranteeing 3 to 10 million paired-end reads per sample in the case of bacterial libraries and 10 to 25 million paired-end reads in the case of human origin libraries. RNA isolation, library generation, and RNA sequencing were performed in three independent replicates.

### 2.3 RNA-seq data analysis

For RNA-Seq data analyses, raw sequencing data were processed with Cutadapt v. 2.8. to remove sequencing adapters (27). Quality trimming with Sickle v.1.33 was then applied, allowing 30% quality and a minimal read length of 20 bp. Reads meeting the required criteria were next aligned to appropriate genomes using the Bowtie2 short read aligner (28) in the case of bacterial RNA or with the help of STAR RNA-seq aligner v.2.7 (29). The genome reference for *M. tuberculosis* H37Rv (https://mycobrowser.epfl.ch/releases, accessed on 12^th^ April 2023; NC_000962.3, v.4) was obtained from the mycobrowser database and the human genome GRCh38 was retrieved from the gencode database (https://www.gencodegenes.org/human/, downloaded on 1^st^ Dec 2018). Aligned data format transformations, sorting and indexing were performed with SAMtools v.1.9 (30) and BEDTools v. 2.27 (31) software suite to generate bedgraphs, whenever needed. While human reads were counted into transcript features within the STAR script, HTSeq v.0.13 was applied for bacterial reads counting (32). Sequencing results were visualized using Integrative Genomics Viewer (IGV) (33). Transcriptional changes were estimated with the online Degust RNA-Seq analysis platform with default parameters (http://degust.erc.monash.edu/, originally designed by D.R. Powell (34) or alternatively with help of the iDEP 96 online platform (http://bioinformatics.sdstate.edu/idep96/, accessed on 12^th^ April 2023 (35). Genes (bacteria) or transcripts (human) with a log2 fold change greater than an absolute value of 1.585 (changing three times or more) and a false discovery rate (FDR) of <0.05 were considered differentially expressed in the current study.

### 2.4 Real-time PCR analysis

The qRTPCR technique was applied as a validation experiment of RNA-Seq data. Reverse transcription was performed using SuperScript III First-Strand Synthesis Super Mix (MP Biomedicals, Irvine, CA, USA) and random hexamers. The expression profile of the studied genes (CXCL8, CCL19, CSF2) was analyzed by qRTPCR using TaqMan chemistry and TaqMan™ Universal PCR Master Mix (Thermo Fisher Scientific, Waltham, MA, USA). The total reaction of 20 µl containing 1X TaqMan™ Universal PCR Master Mix, 1X TaqMan Gene Expression Assay (FAM) and 10 ng of cDNA was activated at 50°C for 2 minutes in order UNG incubation, followed by DNA Polymerase activation at 95 for 10 minutes. Next, 40 cycles of denaturation at 95°C for 15 seconds were followed by annealing/extention at 60°C for 1 minute. qRTPCR assays were run in triplicates on a QuantStudio™ 5 instrument (Applied Biosystems, Carlsbad, California, USA) in 96-well plates. Individual TaqMan Gene Assays with verified amplification efficiencies were purchased from Thermo Fisher Scientific and their corresponding product numbers are listed in **Table S1**. The number of tested transcripts was normalized to the glyceraldehyde-3-phosphate dehydrogenase (GAPDH) housekeeping gene and relative fold changes in gene expression in comparison to the control strain were calculated using the delta method (2^-ΔΔCT^).

### 2.5 Milliplex assay

The concentrations of cytokines, namely G-CSF (CSF3), GM-CSF (CSF2), IL-1α, IL-1β, IL-6, IL-8 (CXCL8), IL-12p40, IL-15, IL-27, TNF-α, CXCL10, CCL2, CCL7, CCL3, CCL4, and CCL5, were determined by applying a commercially available kit for the Milliplex® Multiplex assay and Luminex® Instrument (MERK KGaA, Darmstad, Germany) according to the manufacturer’s recommendations. Milliplex® multiplex assays use the proprietary Luminex® xMAP® bead-based multiplex assay platform. Each magnetic MagPlex® microsphere bead is fluorescently coded with one of 500 specific ratios of two fluorophores (each spectrally distinct set is known as a bead region, fluorescent at λex 635 nm). Additionally, analyte-specific capture antibodies are bound to beads of a specific region, and the beads-bound analyte is detected with biotinylated secondary antibodies. The Luminex^®^ instrument detects individual beads by region plus the streptavidin-conjugated R-Phycoerythrin (SAPE) signal, λex 525 nm, indicating the analyte is present. In short, wells of 96-well titration plates were washed with Wash Buffer at room temperature for 10 min with constant shaking using a plate shaker. Then 25 µL of standard or assay buffer or matrix solution or samples of collected culture media were added to appropriate standard and control wells, background and sample wells, background, standard and control wells, and sample wells, respectively. Furthermore, the 25 µL of premixed beads were introduced to each well and all samples were incubated overnight (16-18 h) at 2-8 ^○^C. On the next day, all wells were washed three times with 200 µL Wash Buffer and the immunoreaction was revealed using 25 µL Detection Antibodies and 25 µL Streptavidin-Phycoerythrin solution per well. After 30 min of incubation, all wells were again washed three times, and finally, 150 µL of Drive Fluid was added to each well and the plate was incubated for 5 min with constant shaking. To determine fluorescence values for standard, experimental, control, and background samples Luminex^®^ equipment was employed. The results were analyzed, and median fluorescent intensity parameters were calculated using dedicated Milliplex assay Belysa™ software (MERK KGaA, Darmstad, Germany).

The assay was performed for three independent healthy blood donors and the samples of collected culture supernatants were run in triplicate.

## 3 Results

We have recently reported that *M. tuberculosis* specifically binds hSAA-1 and the opsonization with 5-fold higher, than the physiological concentration of this acute phase protein favoring bacterial entry into human macrophages and increasing the load of intracellularly multiplying and surviving bacteria (24). On the other hand, an early study by Samaha et al. (36) documented elevated levels of hSAA in both the sera and pleural fluids of tuberculosis patients (93 µg/mL), compared to the physiological concentration of this acute protein (1-2 µg/mL). Here, we asked whether the opsonization of tubercle bacilli with hSAA-1 affects the functional response of professional phagocytes to mycobacterial infection. To answer this question, we applied global RNASeq analysis of total RNAs isolated from human macrophages, prepared from buffy coats of healthy blood donors, to compare the transcription profiles of control MDMs with transcriptomes of MDMs infected with tubercle bacilli, MDMs treated with 5-fold higher than physiological concentration of hSAA-1 (15 µg/mL), and MDMs infected with *Mtb* opsonized with hSAA-1 at concentration of 15 µg/ml. Additionally, we evaluated the transcriptional response of nonopsonized and SAA-1 opsonized tubercle bacilli to the intracellular environment of human macrophages.

MDMs isolated from four healthy blood donors treated or not with hSAA-1 and MDMs infected with opsonized or nonopsonized tubercle bacilli were subjected to total RNA isolation and preparation of RNA libraries for sequencing. Satisfactory quality of RNA (RIN≥7), allowing for sequencing library preparation was obtained from MDMs of three blood donors (three repeats for each blood donor) in the case of control macrophages and macrophages treated with hSAA-1 and from four donors for MDMs infected with opsonized and nonopsonized mycobacteria. As expected, the principal component analysis (PCA) showed that the samples of the repeats of uninfected MDMs, as well as MDMs infected with tubercle bacilli, clustered separately of individual donors (**Figure S1**). By identifying the differentially expressed genes (DEGs) in uninfected MDMs of individual patients we found that approximately 500 to 2,000 genes were differentially expressed (minimum fold change 4, FDR cutoff 0.1) between donors (**Table S2**). The comparisons of DEGs in infected MDMs of individual patients, showing the different donors’ responses to the infection, allowed us to find approximately 300 to 1,200 differentially expressed genes (min fold change 4, FDR cutoff 0.1) between donors. Then, based on all obtained sequences (RNASeq), comparative analyses were carried out to identify the functional response of MDMs to the presence of hSAA-1 and infection with hSAA-1-opsonized and nonopsonized *M. tuberculosis*. Principal component analysis (PCA) of all samples showed that the samples from uninfected macrophages clustered separately from the infected cells and the samples collected from the cells treated with hSAA-1 (**Figure S2**). The differential analysis of gene expression comparing all individual samples to untreated MDMs identified 1392 downregulated and 429 upregulated genes in MDMs infected with opsonized or nonopsonized *Mtb* or treated with hSAA-1 (**Figure S3**).

**Figure 1:**
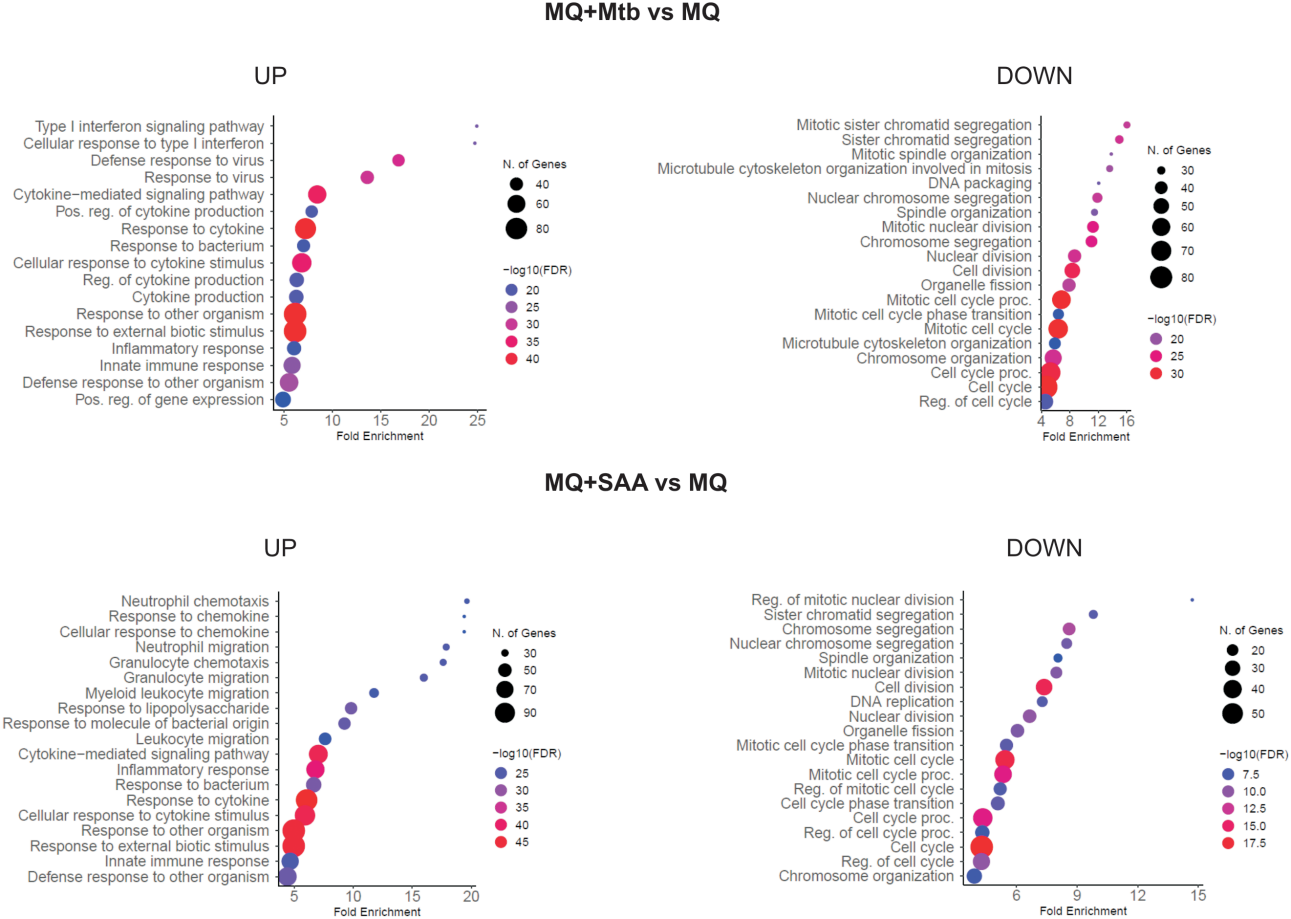
GO biological process enrichment analysis and dotplot charts. MQ represents control macrophages, MQ+Mtb, macrophages infected with *Mtb*; MQ+SAA, macrophages treated with SAA. The diagram was generated by ShinyGO 0.77.

Treatment of MDMs with 5-fold higher than physiological concentrations of hSAA-1, namely 15 µg/mL, for 24 h resulted in alteration of the expression of 684 genes (Log2FC =|1.583|; fold change = |3|; FDR > 0.05). Of these 684 genes, 315 genes were downregulated, and 369 were overexpressed. Furthermore, the infection of MDMs with *Mtb* (MOI 10:1) significantly affected the expression of 633 genes, applying the same criteria of analysis, including 408 downregulated and 225 upregulated genes. Both, treatment with hSAA-1 and infection with *Mtb* led to inhibition of gene expression associated with cell division, cell cycle, nucleosome assembly and organization, and chromosome segregation. On the other hand, the most upregulated genes were classified in the immune response, cytokine-mediated signaling pathway, and response to external biotic stimulus (**Figure 1**). Among the up- and downregulated genes 157 and 191 genes, respectively, were affected by each stimulus.

**Figure 2:**
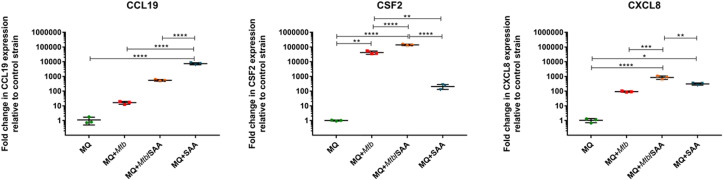
RTPCR (TaqMan) analysis of transcripts for CCL19, CSF2, and CXCL8 genes of MDMs. MQ represents control macrophages, MQ+*Mtb*, macrophages infected with *Mtb*; MQ+*Mtb*/SAA, macrophages infected with *Mtb* opsonized with SAA; MQ+SAA, macrophages treated with SAA. The number of tested transcripts was normalized to the glyceraldehyde-3-phosphate dehydrogenase (GAPDH) housekeeping gene and relative fold changes in gene expression in comparison to the control strain were calculated using the delta method (2-ΔΔCT). The data distribution was evaluated by the Shapiro-Wilk normality test. Statistical analysis was performed by one-way ANOVA with a *post-hoc* Tukey test. *represents *p*<0.05, **p<0.0021, ***p<0.0002, ****p<0.0001.

The RNASeq analyses of MDMs were validated using quantitative RTPCR based on CCL19, highly induced in the presence of hSAA-1, CSF-2, induced after infection with tubercle bacilli, and CXCL8, induced in the presence of either stimuli. The qRTPCR was normalized based on GAPDH housekeeping gene expression (**Figure 2**).

### 3.1 The functional response of human macrophages to the infection with tubercle bacilli

Within the 364 MDM genes upregulated after infection with *Mtb* 223 were annotated as immune-related. The top ten most induced *Mtb-*infected MDM genes contained chemokines and cytokines, namely CXCL8, CCL15, CCL5, IL-1β, and receptor for IL-7 (**Table S3**). As expected the genes encoding other proinflammatory cytokines such as TNFα, IL-6, IL-12, IL-18 and IL-23 were upregulated in MDMs infected with tubercle bacilli, however, the anti-inflammatory cytokines, IL-10 and TGF-β, as well as the antimicrobial peptide cathelicidin were downregulated. As mentioned above, infection with *Mtb* also induced a large number of genes encoding chemokines of the CC and CXC subfamilies, especially chemokines interacting with the receptors CCR5 (CCL3, CCL4, CCL5, CCL8), CXCR1 (CXCL1, CXCL5, CXCL6, CXCL8), CXCR2 (CXCL2, CXCL3, CXCL7), and CXCR3 (CXCL9, CXCL10, CXCL11); however, the receptors CXCR1 and CXCR2 per se were downregulated. The other upregulated cytokines included IL-7, IL-15, CSF2 and their receptors, and CSF3. The upregulated cytokines of the TNF family were TNF, TRAIL, and BAFF, and those of the TGF-β family were INHBA and BMP6. In addition to the abovementioned anti-inflammatory cytokines we also observed the reduced expression of other chemokines and cytokines such as CCL28, CXCL12, and IL-16 (**Figure S4**). Despite the strong overproduction of many chemokines, the chemokine receptors and genes of the chemokine signaling pathway were usually unaffected or downregulated in *Mtb* infected MDMs. The most important exception to this negative regulation is that the Jak-STAT signaling pathway was significantly induced in the infected MDMs.

**Figure 3:**
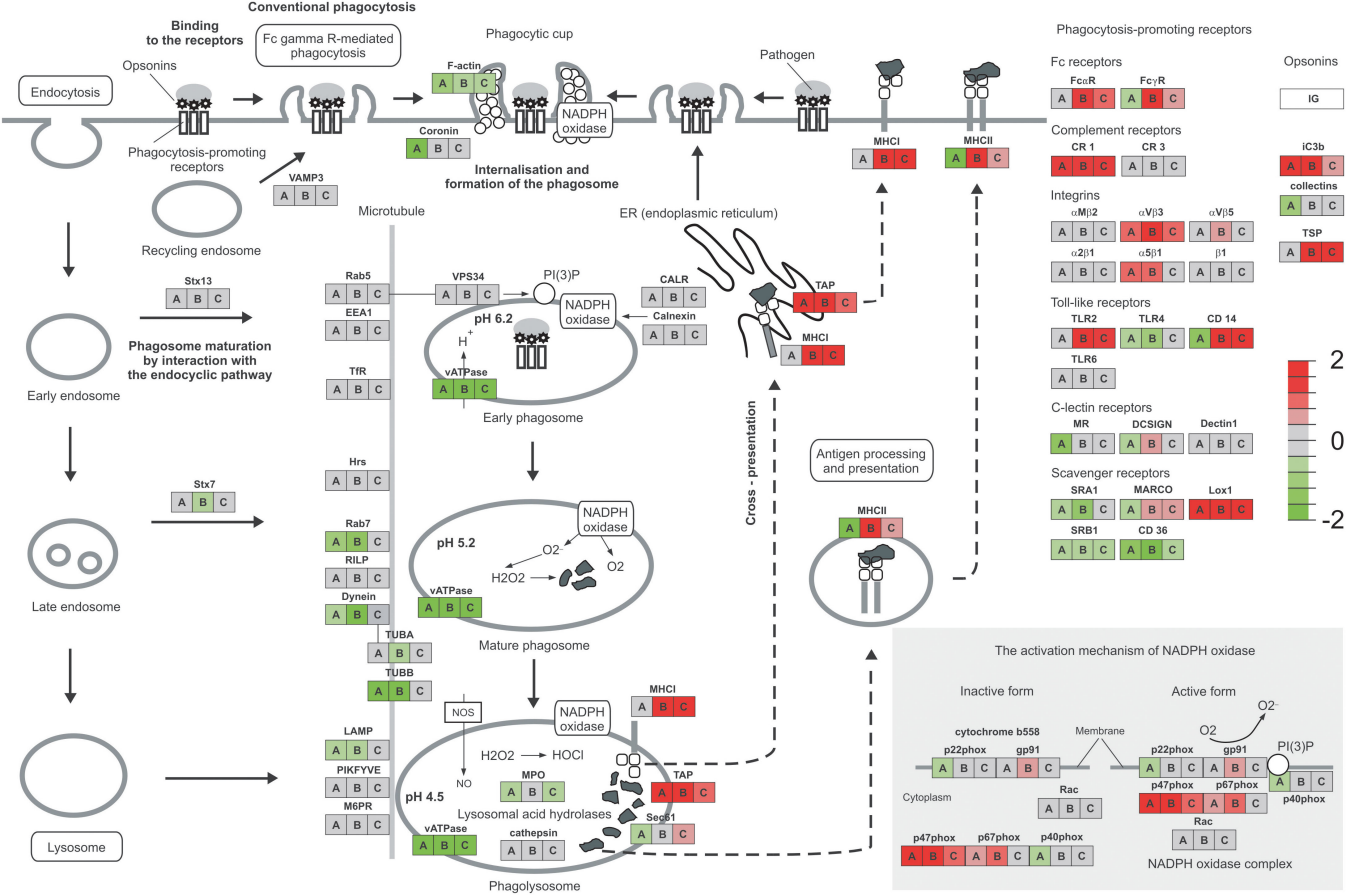
Phagosome and phagocytosis promoting receptors of MDMs. The expression level of genes compared to the control is presented as a heatmap. MDMs infected with *Mtb* are represented by A, hSAA-1 treated MDMs by B, MDMs infected with hSAA-1 opsonized *Mtb* compared to MDMs infected with nonopsonized *Mtb* by C. The analysis was completed based on total RNA sequencing isolated from MDMs of three blood donors (control MDMs and hSAA-1 treated MDMs) or four blood donors (MDMs infected with hSAA-1 opsonized or nonopsonized *Mtb*) in three biological repeats each, completed separately for each comparison using the iDEP.96 platform and presented at each gene as A, B, and C.

The early response to *Mtb* infection is the internalization and intracellular killing of bacilli by alveolar macrophages. Two genes encoding proteins involved in the phagosome formation process, namely coronin and F-actin, were downregulated in infected MDMs. In the early phagosome, weaker expression was observed for vATPase engaged in the phagosome acidification process. Genes encoding major proteins of mature phagosomes, such as Rab7, dynein, and TUBB were also silenced in the infected MDMs. Furthermore, the mature phagosomes are fused to lysosomes and reactive oxygen species are produced to kill intracellularly deposited bacilli. A few genes involved in this process are downregulated in the infected MDMs including major lysosomal membrane protein LAMP, Sec61, lysosomal acid hydrolase MPO and two genes of NADPH oxidase complex p22phox and p40phox. On the other hand, the TAP and p47phox genes are overexpressed. The processed bacterial antigens can be presented by MDMs on the major histocompatibility complex (MHC) molecules class I and class II, to the cells of adaptive immune response. Antigen processing and presentation by MDMs infected with tubercle bacilli can be affected due to the downregulation of MHC class II expression. The phagocytosis process can be facilitated by a number of receptors present on the surface of macrophages. The complement receptor CR1, opsonin iC3b, integrins αVβ3 and α5β1, and scavenger receptor LOX-1 are overexpressed in MDMs infected with *Mtb.* On the other hand, Fc receptor FcyR, Toll-like receptors TLR4, TLR5, and CD14, C-lectin receptors MR and DC-SIGN, scavenger receptors SRA1, MARCO, SRB1, CD36, and collectins are attenuated in infected MDMs. The major phagosome and phagolysosome genes expressed in MDMs and MDMs infected with *M. tuberculosis* are depicted in **Figure 3**.

### 3.2 The functional response of human macrophages in the presence of elevated level of hSAA-1

A total of 269 out of 467 MDM genes upregulated in the presence of an elevated concentration (15 µg/mL) of hSAA-1 were classified as immune-related. The top ten most induced hSAA-1 treated MDM genes contained the same chemokines and cytokines as those induced by *Mtb* infection, such as CXCL8, CCL15, CCL5, IL-1β, and receptors for IL-7 and IL-2 (**Table S3**). We also observed the same pattern of upregulated proinflammatory cytokines (TNFα, IL-6, IL-12, IL-18, and IL-23) and downregulated anti-inflammatory cytokines (IL-10, TGFβ, and antimicrobial peptide cathelicidin) in MDMs treated with hSAA-1 or infected with tubercle bacilli. In the presence of hSAA-1 MDMs induced very similar, but not identical, patterns of genes encoding chemokines of the CC and CXC subfamilies. Additionally, apart from chemokines interacting with the receptors CCR5 (CCL3, CCL4, CCL5, CCL8), CXCR1 (CXCL1, CXCL5, CXCL6, CXCL8), CXCR2 (CXCL2, CXCL3, CXCL7), and CXCR3 (CXCL9, CXCL10, CXCL11) which were induced by both stimuli, hSAA-1 also induced the chemokine ligand of the CXCR5 receptor, namely CXCL13. The receptors CXCR1 and CXCR2 were downregulated, and the receptor CXCR5 was upregulated. The other cytokines upregulated in the presence of hSAA-1 included IL-7, IL-15, CSF2 and their receptors, and CSF3. The upregulated cytokines in the TNF family were TNF, VEGI, and CD70, and those in the TGF-β family were INHBA and BMP6. The downregulated cytokine pattern in hSAA-1 treated or *Mtb* infected MDMs was also very similar with a few additional cytokines downregulated in the presence of hSAA-1 (e.g., LEP, IFNA, OX-40 L, BMP2) (**Figure S5**). MDMs treated with hSAA-1 presented strong overproduction of many chemokines and only a few upregulated chemokine receptors such as CXCR5 and CCR7. In the downstream chemokine signaling pathway, hSAA-1 induced Jak-STAT, IκB, and NFκB genes. The main difference between hSAA-1 treatment and *Mtb* infection of MDMs in the internalization and phagosome formation process is downregulation of coronin observed only after *Mtb* infection. The acidification of phagosomes is affected by vATPase downregulation in the presence of both stimuli. In contrast to infection with *Mtb* in which MHC class II was downregulated, MHC class I and II were significantly overexpressed in the presence of hSAA-1, indicating the role of this stimulus in the induction of antigen processing and presentation. The other significant difference between both stimuli was the MDM downregulation of lysosomal acid hydrolases and genes encoding some subunits of the NADPH oxidase complex, exclusively after infection of MDMs with *Mtb*. Additionally, except for p47phox and p67phox, treatment with hSAA-1 also induced the expression of gp91, the other component of NADPH oxidase. Different transcriptional responses to tubercle bacilli infection and treatment with hSAA-1 were also observed for genes encoding phagocytosis promoting receptors. hSAA-1 induced the expression of Fc receptors (FcαR, FcyR), which were unaffected (FcαR) or downregulated (FcyR) after *Mtb* infection. The complement receptor CR1, opsonin iC3b, integrins αVβ3 and α5β1, and scavenger receptor LOX-1 were overexpressed in MDMs treated with hSAA-1 or infected with *Mtb.* However, exclusively MDMs treated with hSAA-1 also induced genes coding for thrombospondin (TSP), Toll-like receptor TLR2, CD14 (downregulated in infected MDMs), C-lectin receptor DC-SIGN and scavenger receptor MARCO (**Figure 3**).

### 3.3 hSAA-1 enhances the functional response of MDMs to *M. tuberculosis* infection

**Figure 4:**
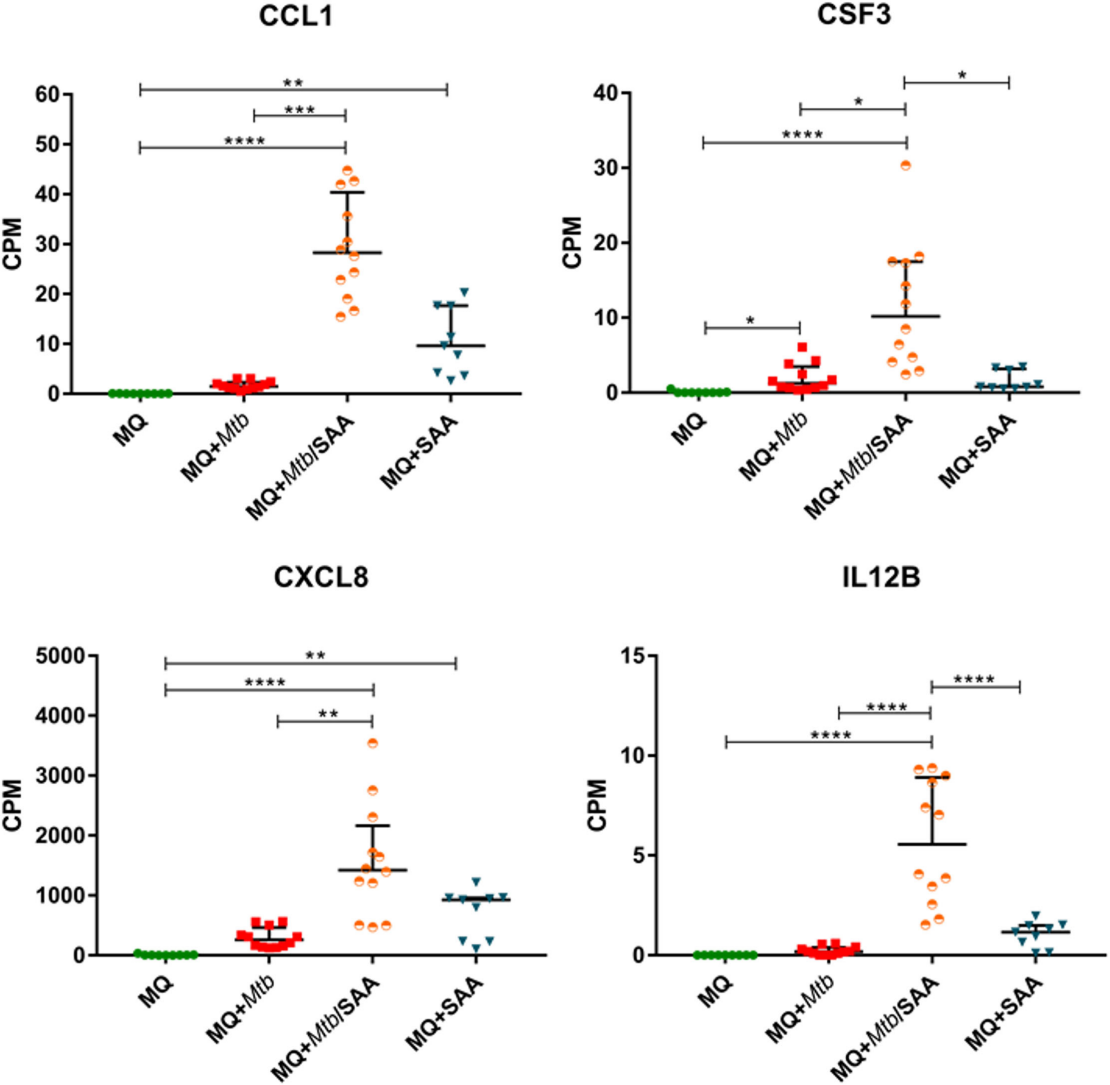
MDM gene expression of selected cytokines. CPM (counts per million) analysis of transcripts for selected cytokines in control MDMs (MQ), MDMs infected with nonopsonized *Mtb* (MQ+Mtb), hSAA-1 opsonized *Mtb* (MQ+Mtb/SAA), and MDMs treated with hSAA-1 (MQ+SAA). The assay was performed for three (MQ, MQ+SAA) or four (MQ+Mtb, MQ+Mtb/SAA) independent healthy blood donors in three biological repeats each. Statistical analysis was performed by nonparametric data distribution, which was evaluated by the Shapiro-Wilk normality test. Furthermore, statistical analysis was performed by Kruskal-Wallis one-way ANOVA with *post-hoc* Dunn’s test or one-way ANOVA with *post-hoc* Tukey’s test (IL12B). *represents *p*<0.05, **p<0.0021, ***p<0.0002, ****p<0.0001.

Since hSAA-1 opsonizes tubercle bacilli, we asked whether such opsonization modulates the functional response of MDMs to the infection with *Mtb*. The *in vitro* opsonized bacilli were used for infection of MDMs, and then 24 h postinfection total RNA was isolated and sequenced. Comparing RNASeq analysis for MDMs infected with nonopsonized and hSAA-1-opsonized *Mtb*, 96 genes with significantly affected expression (FDR=0.05, fold change=3, Log2FC=1.583) were detected, including 90 overexpressed genes. The top ten most induced genes of MDMs infected with opsonized bacilli, compared to MDMs infected with nonopsonized bacilli, contained interleukins IL-12β, IL-1β and CCL1 chemokine, and receptor for IL-2 (**Table S3**). Most chemokines of the CC and CXC subfamilies overexpressed in MDMs treated with hSAA-1 or in MDMs infected with tubercle bacilli presented synergistic effects in MDMs infected with opsonized bacilli resulting in elevated levels of transcripts (e.g., CCL3, CCL4, CXCL1); however, CCL19 and CCL7 presented elevated levels of transcripts exclusively in the presence of hSAA-1 (**Figure 4**, **Figure S6**).

On the other hand, CXCL11 was downregulated in the presence of opsonized bacilli compared to MDMs infected with nonopsonized *Mtb*. The downregulation dependent on hSAA-1 opsonization of tubercle bacilli was also determined for genes encoding the IL-9 receptor, OX40 ligand (OX40L) of the TNF family and growth differentiation factor GDF9. The hSAA-1 dependent upregulation was identified for a few genes encoding cytokines (IL-10, lymphotoxin β - LTβ) and cytokine receptors (IL-12R, IL-17R, IL-10R, activin A receptor like-protein 1 -ACVRL1), (**Figure S6**). The opsonization of tubercle bacilli with hSAA-1 also affects the expression of a few genes encoding proteins involved in phagosome formation, maturation and fusion to lysosomes. If MDMs are infected with hSAA-1-opsonized bacilli, the F-actin, vATPase and myeloperoxidase (MPO) genes are attenuated compared to infection with nonopsonized bacilli; however, genes encoding transporters associated with antigen processing (TAP) and MHC class I and II molecules are significantly upregulated. The opsonized bacilli also upregulate a component of the NADPH oxidase complex, namely p47phox. Within the phagocytosis-promoting receptors, the opsonization of *Mtb* with hSAA-1, led to the upregulation of Fc receptors (FcαR, FcyR), complement receptor CR1, integrin αVβ3, Toll-like receptor TLR2, CD14, scavenger receptor LOX-1, TSP and opsonin iC3b. On the other hand, hSAA-1 inhibits the expression of scavenger receptors SRB1 and CD36 (**Figure 3**).

### 3.4 The response of human professional phagocytes to hSAA-1 and *M. tuberculosis* infection is detectable at the protein level

**Figure 5:**
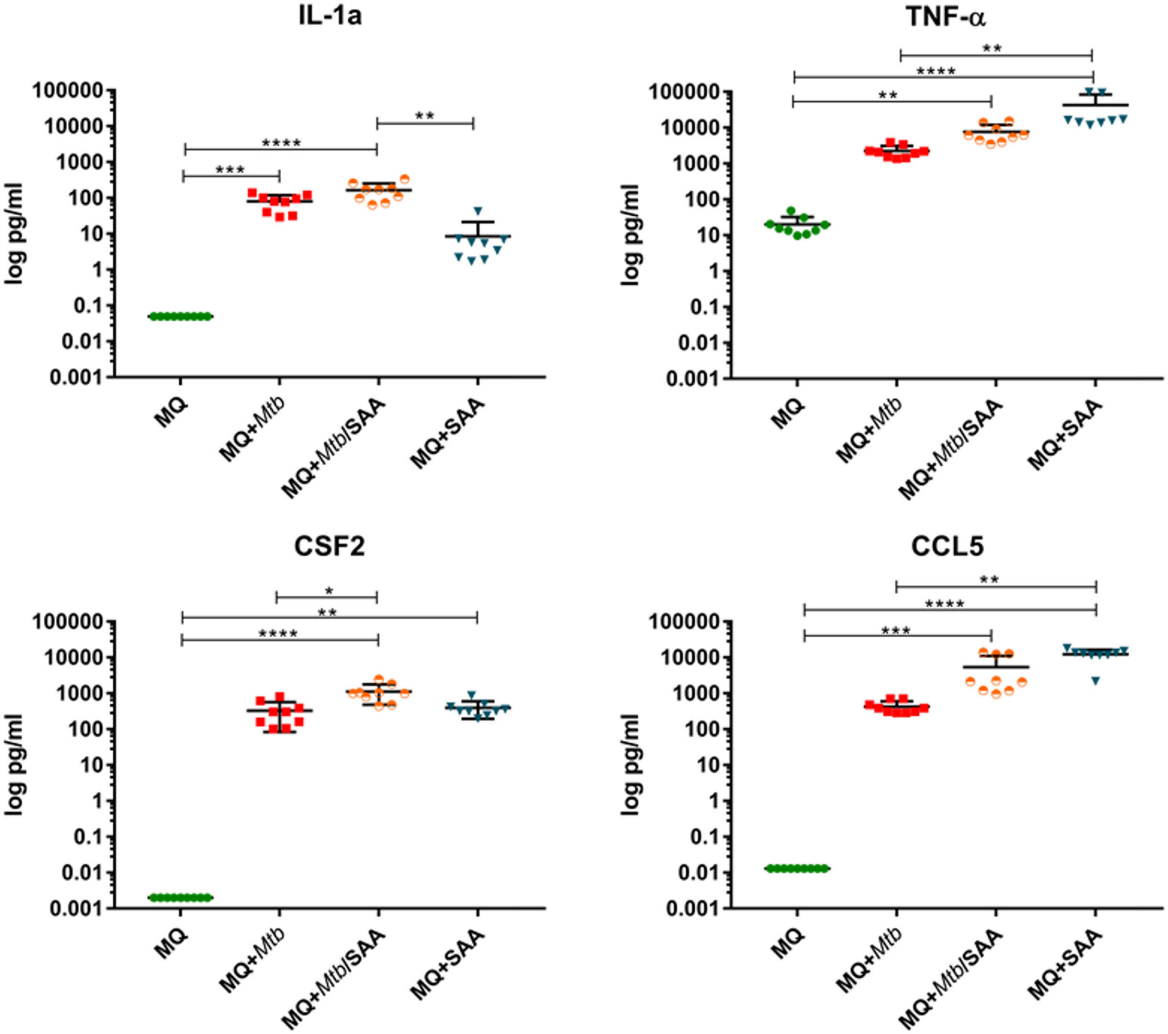
The concentrations of selected cytokines were determined using the Milliplex system. The protein level was assessed in the cell supernatant of control MDMs (MQ), MDMs infected with nonopsonized *Mtb* (MQ+*Mtb*), hSAA-1 opsonized *Mtb* (MQ+*Mtb*/SAA), and MDMs treated with hSAA-1 (MQ+SAA). The assay was performed for three independent healthy blood donors and the samples of collected culture supernatants were run in triplicate. The data distribution was evaluated by the Shapiro-Wilk normality test. Furthermore, statistical analysis was performed by Kruskal-Wallis one-way ANOVA with *post-hoc* Dunn’s test. *represents p<0.05, **p<0.0021, ***p<0.0002, ****p<0.0001.

The functional response of MDMs to hSAA-1 and/or tubercle bacilli was verified for selected cytokines at the protein level by applying the Milliplex system. The concentrations of protein were determined in the culture medium of MDMs, MDMs treated with 5-fold higher than physiological concentration of hSAA-1 (15 µg/mL), MDMs infected with tubercle bacilli, and MDMs infected with *Mtb* opsonized with hSAA-1. Based on the RNASeq results, sixteen cytokines were selected for the analysis and included cytokines induced in the presence of either stimulus (TNF-α, CCL5, CXCL8, CCL4, IL-15, IL-1β, IL-6, CCL3, CSF3), induced exclusively by hSAA-1 (CCL3, IL-12β, CCL2), induced exclusively by infection with *M. tuberculosis* (CSF2, IL-27, CXCL10), and induced only in the presence of both stimuli (CCL7), (**Table S3**). Cytokines were identified in the culture medium of control MDMs at various concentrations. The most abundant cytokines detected at concentrations over 1000 pg/mL in the culture medium of control MDMs were CCL2 and CXCL8. On the other hand, the concentrations of CCL5, CSF2, CXCL10 and IL-12β were below 1 pg/mL. The other cytokines produced by control MDMs were at concentrations >1<10 pg/mL (IL-15, IL-1β, CSF3), >10<100 pg/mL (CCL4, IL-27, IL-6, CCL3), and >100<1000 pg/mL (CCL7). The treatment of MDMs with hSAA-1 increased the concentrations of cytokines over 9.5 ng/mL (IL-6, CXCL8, CCL2, CCL3, CCL5, TNF-α), 1 ng/mL (CSF3, CSF2, IL-12B, CXCL10, CCL7, CCL4), 100 pg/mL (IL-27), 10 pg/mL (IL-15) and >1<10 pg/mL (IL-1α, IL-1β). The lowest 4-fold increase in the cytokine concentration after treatment with hSAA-1 was noted for interleukin IL-27, and the most abundant increase in the concentrations was observed for CCL5, CSF2, IL-12β, and CXCL10. Infection of MDMs with tubercle bacilli led to an increase in the concentration of most tested cytokines except CCL2 and IL-27. The highest concentrations, over 9.5 ng/mL were detected for CXCL8 and CCL3. The culture medium of MDMs infected with *Mtb* also contained over 1 ng/mL of CSF3, CCL2 (decrease in concentrations compared to control MDMs), CCL7, CCL4 and TNF-α. The cytokines IL-6, IL-27, and CCL5 were detected at concentrations over 100 pg/mL, CSF2, IL-1α, IL-1β, IL-12β and CXCL10 at concentrations above 10 pg/mL, and IL-15 at concentrations below 10 pg/mL. The most significant, at least 100-fold, increase in the concentrations was detected for CSF2, CSF3, IL-1α, CCL3, CCL4, CCL5 and TNF-α. The infection of MDMs with *Mtb* opsonized with hSAA-1, compared to the infection with nonopsonized bacilli, led to at least a 2-fold increase in the concentrations of CSF2, CSF3, IL-1α, IL-1β, IL-6, IL-12, CCL15, and TNF-α with the most potent 10-fold increase observed for CCL5. The concentrations of other tested cytokines (CXCL8, IL-15, IL-27, CXCL10, CCL2, CCL7, CCL3, CCL4) were at similar levels in the media of MDMs infected with opsonized and nonopsonized bacilli (**Figure 5**, **Figure S7**).

### 3.5 The functional response of tubercle bacilli to the intracellular environment of human macrophages

**Figure 6:**
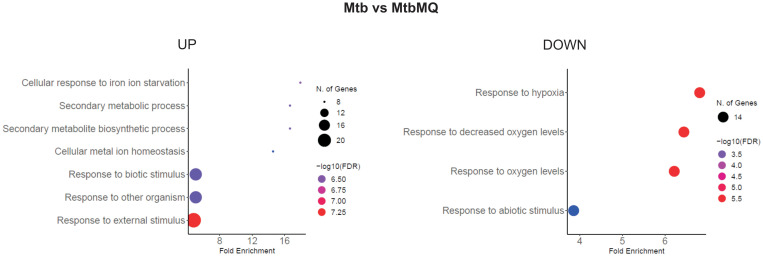
GO biological process enrichment analysis of *Mtb* intracellular environment-induced changes. Mtb represents control bacilli, MtbMQ represents *Mtb* isolated from MDMs. The diagram was generated by ShinyGO 0.77.

Human monocyte-derived macrophages obtained from buffy coats of four healthy blood donors were infected with *Mtb* opsonized or nonopsonized with hSAA-1. The bacilli were released from phagocytes 24 h postinfection and used for RNA isolation and sequencing. The control bacilli were incubated in the same media without MDMs for the same time. The bioinformatic analysis of RNASeq data shows the global response of bacilli to the intracellular environment of macrophages and the potential modulation of this response by hSAA-1. Based on our selection criteria for differentially expressed genes (Log2FC = |1.583|; fold change = |3|; false discovery rate (FDR) of <0.05), global transcriptional analysis of tubercle bacilli released from macrophages identified 302 genes presenting significantly changed expression levels. Of these 139 genes were upregulated, while 163 were downregulated. Among the most enriched GO pathways in DEGs of tubercle bacilli residing in human macrophages the cellular response to iron starvation, metal ion homeostasis, response to hypoxia and response to other organisms or abiotic stimuli were identified (**Figure 6**).

**Figure 7:**
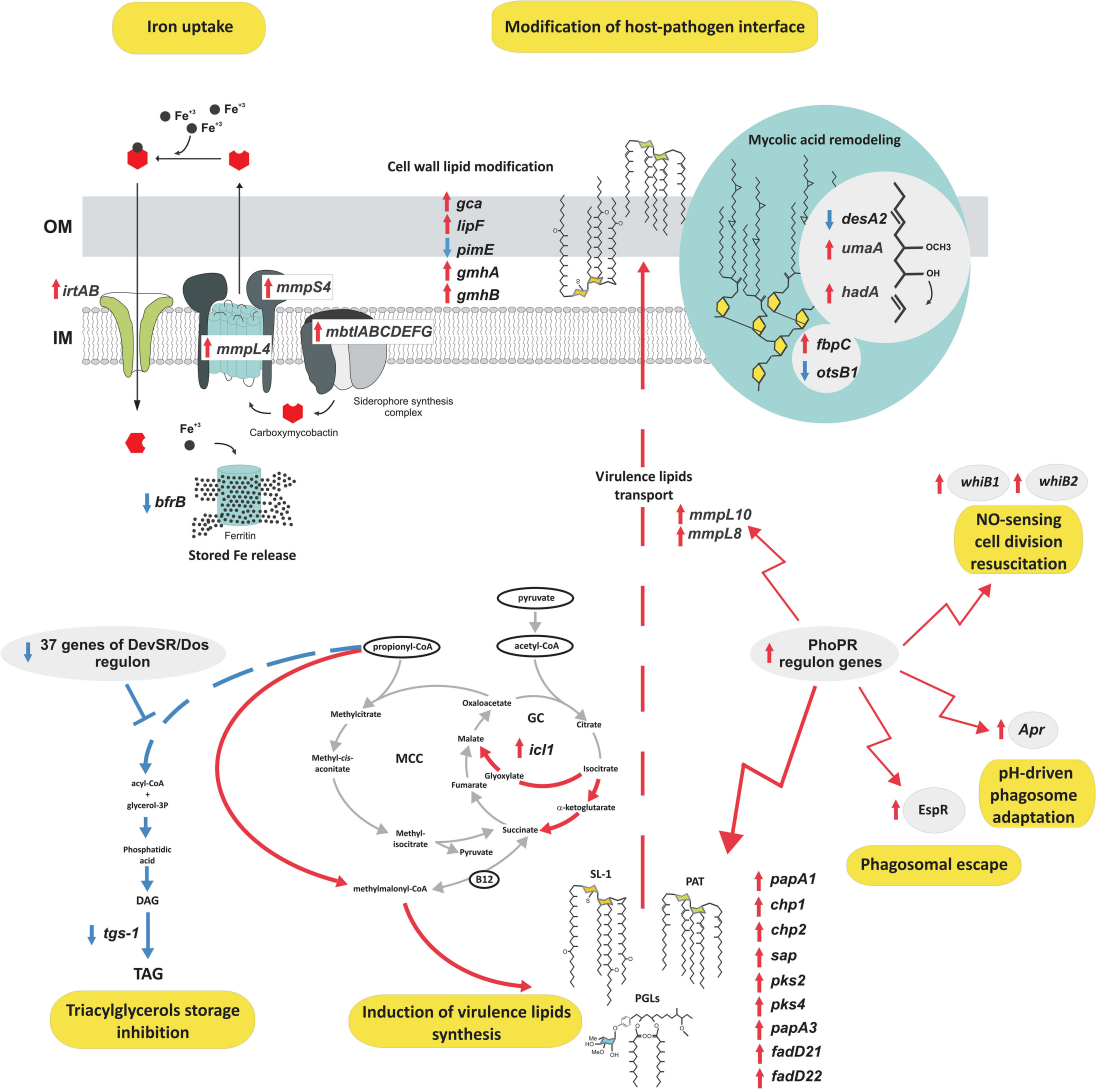
The functional response of tubercle bacilli to the intracellular environment of MDMs. Red arrows, upregulation; blue arrows, downregulation; IM, inner membrane; OM, outer membrane; SL, sulfolipids; PAT, polyacylated trehalose; PGL, phenolic glycolipids.

A massive increase in expression of the whole IdeR-regulated machinery of siderophore-based iron acquisition (MbtA-G, IrtAB, MmpL4/S4, HisE, PPE37, Rv3403c, Rv3839) (37) and critical for survival under host-mediated stress regulators of siderophore synthesis (HubB) (38) confirms that tubercle bacilli extensively prevent iron sequestration inside macrophages. The intracellular environment of MDMs increases the expression of a whole set of virulence regulators (EspR, Lsr2, WhiB1, WhiB2) and proteins that directly coordinate the inhibition of phagosomal maturation (SapM, EsxH, AprB), phagosomal rupture, biofilm formation, pH sensing, escape from the phagolysosome or modulate the T-cell response and secretion of immunomodulatory PE/PPE proteins (39–41). Moreover, a significant change in the expression of secretion systems such as SEC, TAT, ESX-1, and ESX-3 involved in host-pathogen encounters, promoting growth in macrophages and inhibiting the host immune response was also identified (42, 43). Overall, many identified DEGs represent regulons of two-component signal transduction systems that are strictly involved in response to hypoxia, NO level, low pH, and adaptation to the increasing level of CO_2_ (PhoPR, DevR-DevS, TrcRS, KdpDE) including the significant overrepresentation of PhoPR-regulated genes, within upregulated CDSs and an almost complete set of DevR-DevS system genes, within downregulated CDSs. PhoPR regulates approximately 80 to 150 genes essential for virulence and complex lipid biosynthesis (40). Indeed, a closer look at the lipid metabolism pathways upregulated upon infection reveals significant induction of almost a complete array of genes encoding the synthesis and transport of sulfolipids, acylated trehaloses, or phenolic glycolipids – lipids playing crucial roles in mycobacterial virulence (44) (**Figure 7**).

Along with increased virulence lipid synthesis, the data also suggests induction of the cell wall mycolic acid remodeling program and modification in arabinogalactan mycolylation or lipid glycosylation patterns. Conversely, we observed extremely strong downregulation of the DevR-DevS-regulated triacylglycerol synthesis gene – *tgs-1* and the absence of any transcriptional response within lipid catabolism or pathways ameliorating lipid degradation metabolites such as the methylmalonyl pathway or methylcitrate cycle, with the exception of increased isocitrate lyase - *icl*1 gene expression also involved in TCA and the glyoxylate cycle. To analyze the specific effect of hSAA-1 opsonization on the transcriptional profile of tubercle bacilli infecting human macrophages we compared the transcriptional response of opsonized and nonopsonized *Mtb* to the intraphagosomal environment. The previously identified *in vitro* response of *Mtb* to SAA was not considered in this analysis (24). Principal component analysis (PCA) demonstrated that transcriptomic responses of the opsonized and nonopsonized bacteria are clustered (**Figure S8**). Indeed, their response to the intraphagosomal environment was almost identical in most aspects mentioned above, however, a detailed comparison of DEGs between these two datasets allowed the identification of 11 genes whose infection-induced expression change was found to be specific only for the bacilli opsonized with hSAA-1 (**Table S4**). Conversely, 28 genes differentially expressed in nonopsonized bacilli infecting MDMs displayed unchanged expression in *Mtb* opsonized with hSAA-1 (**Table S4**). Most of the 39 mentioned genes represent membrane/transmembrane or cell wall-associated/secreted proteins, transcriptional regulatory proteins, elements of efflux systems, or toxin-antitoxin components. The common feature is their presumed exposition in the host-pathogen interface, reported antigenicity, involvement in virulence, stress adaptation, or metal ion/drug efflux.

## 4 Discussion

Alveolar macrophages (AMs) and interstitial macrophages (IMs) are two major macrophage subsets in the lungs. AMs located in the alveolar space of the lung present higher engulfment capacity against antigens and pathogens and constitute the first line of defense. AMs play a central role in homeostasis, tissue remodeling and during pathogen infection and inflammation, and produce various cytokines such as TGF-β, IL-6, and type I interferons (45). IMs form a heterogeneous population in the parenchyma, between the microvascular endothelium and alveolar epithelium, engulf bacteria and secrete IL-1, IL-6, IL-10, and TNF-α (46). The capacity of macrophages is modulated by a number of factors including SAA1, which is highly upregulated in response to inflammation, as well as, during tuberculosis. MDMs, which we used in our study, are considered the current best alternative experimental model to alveolar macrophages containing two different cell types, namely tissue-resident alveolar macrophages (TR-AMs) and monocyte-derived alveolar macrophages (MdAMs). Although TrAMs and MdAMs possess different origins and some transcriptional characteristics, both subsets of these cells are important in pathogen clearance, initiation and resolution of inflammation, and lung tissue recovery. Additionally, MdAMs, originating from blood monocytes recruited to the lungs by cytokines and chemokines produced by TrAMs and other cells, could also participate in the restoration of a depleted pool of TrAMs (4, 47). However, despite the cooperation of TrAMs and MdAMs during *Mtb* infection, these two populations of lung macrophages possess different phenotypes. While MdAMs are polarized to the M1 type of macrophages, TrAMs cells exhibit characteristics of both classically and alternatively activated M1 and M2 macrophages, respectively (48). Despite the high level of expression of mannose receptors, type A scavenger receptors, TLR9 receptor, and high phagocytic activity, the suppressor properties of these cells are indicated, due to the low activity of the TLR2 receptor, CD80 and CD86 costimulatory molecules, and weak bactericidal activity, and limited synthesis of reactive oxygen compounds compared to peripheral blood monocytes and neutrophils (45, 49–52). In addition, the reduced ability of TrAMs to present antigens, as well as their inhibitory effect on the activity of dendritic cells and lymphocyte activation, are also emphasized (53–56). Considering the above-mentioned differences between MdAMs and TrAMs, it can be cautiously noted that the response of these two types of macrophages to hSAA-1 stimulation and infection by non-opsonized and hSAA-1-opsonized tubercle bacilli may differ, however, a reliable response requires further research.

Here, we analyzed the functional response of MDMs to a 5-fold higher than physiological concentration of hSAA-1 (15 µg/mL) in comparison to the response of MDMs to *Mtb* infection. We found that at the cytokine gene expression level the phagocyte response to both stimuli was very similar, with abundant expression of genes coding for pro-inflammatory cytokines, as well as chemokines CXCL8, CCL15, CCL5, and downregulation of anti-inflammatory cytokines. The only cytokines induced by hSAA-1 but not by tubercle bacilli were chemokines CCL19 and CXCL13. On the other hand, the TNF-family cytokine TRAIL (TNF-related apoptosis-inducing ligand) was upregulated after infection but not after hSAA-1 treatment. CCL19 was described as a strong chemotactic factor for B cells and various subpopulations of T lymphocytes (57), and CXCL13 is selectively chemotactic for B cells and elicits its effects by interacting with chemokine receptor CXCR5 (58), which was also upregulated as a result of hSAA-1 stimulation. TRAIL is a protein that functions as a ligand that induces caspase-8-dependent apoptosis. In cells expressing DcR2 (Decoy receptor 2), TRAIL binding activates NFκB, leading to transcription of genes known to antagonize the death signaling pathway and/or to promote inflammation (59).

The analysis of the functional response of MDMs to the hSAA-1 opsonized bacilli has shown that the response to both stimuli is not only very similar but also has a synergistic effect for many genes encoding cytokines (**Table S5**). However, in some cases, the opsonization of *Mtb* with the human acute phase protein had the opposite effect compared to that induced by each stimulus individually. MDMs infected with the opsonized tubercle bacilli, compared to MDMs infected with the nonopsonized pathogen, downregulated CXCL11 and upregulated IL-10 genes which were upregulated and downregulated in the phagocytes treated by hSAA-1 and infected with nonopsonized *Mtb*, respectively. CXCL11 is chemokine strongly induced by IFN-γ and IFN-β, chemotactic for activated T cells (60). IL-10, known as human cytokine synthesis inhibitory factor (CSIF), is an anti-inflammatory cytokine with multiple, pleiotropic effects related to immunoregulation and inflammation. It downregulates the expression of Th1 cytokines, MHC class II antigens, and costimulatory molecules on macrophages. It also enhances B-cell survival, proliferation, and antibody production. IL-10 can block NF-κB activity and is involved in the regulation of the JAK-STAT signaling pathway induced in both MDMs treated with hSAA-1 and infected with *Mtb*. IL-10 induces STAT3 signaling via phosphorylation of the cytoplasmic tail of the IL-10 receptor (61). A similar response to infection with virulent *M. tuberculosis* H37Rv, compared to avirulent *M. tuberculosis* H37Ra and *M. bovis* BCG, was reported for the THP-1 human macrophage cell line. Authors observed significant increase in the expression of IL-1β, TNF-α, CCL3, CCL4, CSF2 and downregulation of IL-10 and CCL2. An increased gene expression profile was also observed for chemokines such as CXCL1, CXCL2, CXCL3, CXCL8, CCL3, and CXCL4 engaged in the recruitment of polymorphonuclear cells, such as neutrophils. Of the listed cytokines, in our MDM model, we observed a different response only for the chemokines CCL2 presenting a chemotactic activity for monocytes and basophils, and CXCL4 a strong chemoattractant for neutrophils, fibroblasts, and monocytes, both showing increased expression in the presence of the nonopsonized and, in particular, opsonized mycobacteria. The infection of THP-1 with virulent bacilli selectively induced IL-23 rather than IL-12 and the enhanced expression of IL-17RB and IL-17RE receptors indicating the Th17-dominated inflammatory T-cell response (62). However, infected MDMs induced the expression of both IL-23 and IL-12 and significantly downregulated the expression of IL-17 receptors. Neither infection with *Mtb* nor treatment with hSAA-1 induced the expression of Th2 cytokines in MDMs, namely IL-4 and IL-13, which polarize macrophages to an M2 activation status (63–65). At least at the time of analysis, 24 h postinfection, MDMs presented proinflammatory M1 polarization.

The response of MDMs to hSAA-1 and *Mtb* infection revealed more differences in the gene expression encoding players in phagosome formation, maturation, and phagolysosome fusion. Although both factors inhibit phagosome acidification through downregulation of vacuolar ATPase (vATPase) (66), genes encoding coronin, lysosomal acid hydrolases, Sec61 translocating antigens from the endosomal compartments to the cytosol (67) and some components of the NADPH oxidase complex were downregulated exclusively in *Mtb* infected MDMs. On the other hand, both stimuli enhanced the expression of TAP, which is involved in antigen processing and translocation; however, hSAA-1 exclusively affects antigen processing and presentation by overexpression of MHC class I and II molecules, with the latter being downregulated in *Mtb* infected MDMs. Manipulation and inhibition of antigen processing and presentation are considered highly evolved evasion strategies of *Mtb* resulting in its intracellular persistence in the hostile microenvironment of macrophages and in an altered specific T-cell adaptive immune response. One of these pathways disrupted by tubercle bacilli is antigen processing and presentation served by MHC II molecules. After infection, *Mtb* can affect the expression of MHC class II in macrophages by blocking the fusion of phagosomes with lysosomes and phagosome acidification (11, 68). Inhibition of phagolysosome fusion could potentially be a result of the functional activity of *Mtb* proteins (e.g. kinase G) and is indicated as an important mechanism allowing tubercle bacilli to avoid the activity of lysosomal hydrolases (69), which are also downregulated by the pathogen. Consequently, the lack of development of the phagolysosome compartment disrupts intracellular processing of the bacterial antigens and loading of the peptides onto MHC class II molecules. The diminished phagosome acidification level could also be related to the downregulation of vacuolar proton-ATPase expression observed in our study. The studies performed in other laboratories revealed selective inhibition of incorporation or retention of intact vATPase by the mycobacterial phagosome, which could result in the arrest of the phagosome acidification and bacterial antigen processing and presentation to the CD4^+^ T lymphocytes responsible for the development of an effective adaptive immune response (70). Surprisingly, the expression of the gene encoding vATPase was also downregulated by SAA1; however, it did not lead to the downregulation of the expression of MHC class II, which was overexpressed in SAA1-stimulated macrophages. In addition to manipulating MHC II-dependent antigen processing and presentation, *Mtb* can also affect the MHC class I pathway due to the noted downregulation of Sec61 translocation and upregulation of TAP transporters, which are engaged in the translocation of proteins to the cytosol (71) and phagosomal processing of tubercle bacilli antigens and their loading onto MHC I molecules (72, 73).

Significant differences are also observed in gene expression for phagocytosis promoting receptors in MDMs treated with hSAA-1 or infected with tubercle bacilli. Fc receptors (FcαR, FcyR), Toll-like receptor TLR2 and cell surface receptor and differentiation marker CD14 were exclusively overproduced in the presence of hSAA-1 and downregulated (FcyR, TLR2, TLR4, CD14) during infection. The C-lectin receptor DCSIGN, scavenger receptor MARCO and collectins were downregulated during infection but not in MDMs stimulated with hSAA-1. On the other hand, both stimuli induced complement opsonin iC3b, complement receptor CR1, and scavenger receptor LOX-1, and suppressed TLR4. The immune response to tubercle bacilli is initiated by PRRs including Toll-like receptors, nucleotide-binding domain and leucine-rich repeat-containing receptors (NLRs), C-type lectin receptors (CLRs) and cyclic GMP-AMP synthase (cGAS) (74),. The mouse model revealed that TLR2 recognizes bacterial lipoproteins and lipoglycans, and TLR9 recognizes unmethylated CpG DNA as the most important in the control of *Mtb* infection (75–78). We found that genes encoding TLR2 and CD14 are highly induced in human macrophages treated with hSAA-1 alone or infected with hSAA-1-opsonized tubercle bacilli but are not affected (TLR2) or suppressed (CD14) in macrophages infected with nonopsonized *Mtb*. In contrast, increased CD14 expression was observed in THP-1 cells infected with virulent and avirulent tubercle bacilli (62). As reported, CD14 constitutively interacts with the MyD88-dependent TLR7 and TLR9 pathways and is required for TLR7- and TLR9-dependent induction of proinflammatory cytokines *in vitro* and for TLR9-dependent innate immune responses in mice (79). The cell wall components of tubercle bacilli, such as glycolipids and lipoarabinomannan can be recognized by CLRs including DC-SIGN, mannose receptor (MR) or Dectin-1 (80–83). DCSIGN and MR were downregulated in MDMs infected with *Mtb* and not affected (MR, Dectin-1) or slightly induced (DCSIGN) in hSAA-1-treated human macrophages. Among the scavenger receptors, LOX-1 was highly induced in MDMs both during mycobacterial infection and hSAA-1 stimulation. hSAA-1 also induced expression of the MARCO receptor, which was downregulated during infection, similar to the SRA1, SRB1, and CD36 receptors. LOX-1 is involved in the accumulation of oxidized low-density lipoprotein particles (OxLDL) within vascular cells. LOX-1 mediates OxLDL endocytosis via a clathrin-independent internalization pathway. Transgenic animal model studies have shown that LOX-1 plays a significant role in atherosclerotic plaque initiation and progression. LOX-1 endocytosis is also potentially important in immune surveillance as it has been shown to regulate antigen presentation by MHC class I and II molecules (84). Elevated surface expression of the type 1 scavenger receptors CD36 and LOX-1 was also reported for guinea pig macrophages infected with *Mtb*, which facilitated the uptake of oxidized host macromolecules including OxLDL (85).

We also analyzed the functional response of MDMs to hSAA-1 and *Mtb* infection at the protein level. Most of the selected cytokines were upregulated in MDMs treated with hSAA-1, except IL-27 and CCL7. The genes encoding these cytokines were also classified as uninduced in RNASeq analysis. On the other hand, CXCL10 and CSF2 were also uninduced at the RNA level but overproduced at the protein level. The discrepancy can result due to the cut off value (fold change=3) used in RNASeq analysis, since genes for CXCL10 and CSF2 were upregulated, however, to a lower extent (fold change 1.2 and 1.6, respectively). Infection of MDMs with non-opsonized bacilli induced, at least 10-fold, the synthesis of most tested cytokines except IL-15, IL-27, CCL2 and CCL7. Chemokines CCL2 and CCL7 were also uninduced at the RNA level; however, IL-15 and IL-27 genes were significantly upregulated in RNASeq. The IL-27 concentration was quite high in control, uninfected MDMs (>70 pg/mL) and increased after infection by approximately 50% (106.5 pg/mL). The amount of IL-15 also increased by approximately 50% after infection with *Mtb*, which is significantly less than the mRNA level, suggesting posttranscriptional control. We did not observe much difference in the tested cytokines in MDMs infected with the opsonized and nonopsonized bacilli. The only cytokine with a concentration that increased more than 10-fold (from 206 to 2705 pg/mL) when the opsonized bacilli were used, was the chemokine CCL5; however, the concentrations of other cytokines (CSF3, CSF2, IL-1α, IL-1β, IL-6, IL-12, TNF-α) increased to a lesser extent. CCL5 was also upregulated at the mRNA level in MDMs after infection with opsonized bacilli, even though memory CD8^+^ T-cells have a large amount of preformed CCL5 mRNA in the cytoplasm and chemokine secretion was reported to be dependent only on translation (86). CCL5 is characterized by proinflammatory activity and chemotactic activity for T cells, eosinophils, basophils, monocytes, natural killer (NK) cells, dendritic cells, and mastocytes (87).

During infection, *Mtb* must adapt to changing conditions. A global adaptive response resulting from changes in available carbon sources, pH, oxygen access, is essential in macrophages, granulomas and during the reactivation process. Transcriptomic profiles of *Mtb* reactivating from hypoxia-induced non-replicating persistence revealed a global gene expression reprogramming with number of up-regulated transcription regulons and metabolic pathways, including those involved in metal transport and remobilization, second messenger-mediated responses, DNA repair and recombination, and synthesis of major cell wall components (88). During infection, *Mtb* must adapt to changing conditions. A global adaptive response resulting from changes in available carbon sources, pH, oxygen access, is essential in macrophages, granulomas and during the reactivation process. Transcriptomic profiles of *Mtb* reactivating from hypoxia-induced non-replicating persistence revealed a global gene expression reprogramming with number of up-regulated transcription regulons and metabolic pathways, including those involved in metal transport and remobilization, second messenger-mediated responses, DNA repair and recombination, and synthesis of major cell wall components of *Mtb* located inside macrophages for 24 h. The analysis of DEGs representing enriched metabolic clusters in bacilli isolated from MDMs clearly demonstrates that at the early stage of infection, *Mtb* activates at least two main virulence strategies: immune modulation, and phagosomal survival and rupture. It is, in turn, accompanied by unchanged or downregulated expression within pathways specific for prolonged infection, granuloma formation, and dormancy (e.g. DevR-DevS regulon). The *Mtb* genes affected by the intracellular environment of MDMs are strictly involved in response to hypoxia, NO level, low pH, adaptation to the increasing level of CO_2_, synthesis of virulence effectors (e.g. PhoPR regulon), and secretion systems such as SEC, TAT, ESX-1, and ESX-3. At this early stage of infection, the results also showed high upregulation of genes for the synthesis and transport of surface-exposed lipids such as sulfolipids, acylated trehaloses, or phenolic glycolipids that constitute the hydrophobic barrier around the bacterium and are also known as modulators of host cell function, acting as highly potent virulence modulators (89). Interestingly, 24 h postinfection, those events are apparently not accompanied by the increased utilization of lipids as the energy source or energy reserve. Neither the cholesterol/fatty acid degradation pathway nor the methylcitrate cycle ameliorating the degradation of lipid metabolites, namely propionyl-CoA, was induced. Moreover, we observed extremely strong downregulation of the DevR-DevS-regulated *tgs-1* gene encoding an enzyme that synthesizes triacylglycerol, a major energy reserve for resuscitation from dormancy (44). This suggests a clear orientation of *Mtb* lipid metabolism during early infection of MDMs toward modification of the lipid host-pathogen interface to modulate the host response and promote survival within the phagosome. In previously published analyses of the *Mtb* transcriptional response to the macrophage environment, the authors suggest rapid remodeling of metabolism to consume lipids, especially cholesterol, and activation of the metylcitrate cycle ameliorating lipid catabolism end-products (90–93). However, our data show that the early stage of infection is not accompanied by any dramatic changes in central carbon and energy metabolism and shows no transcriptomic signs of lipid consumption/storage or nutrient starvation suggesting that macrophages are still abundant in diverse, readily available carbon sources. We also did not find transcriptional signs of bypassing the citric acid cycle oxidative pathway or upregulation of the methylcitrate cycle which together with the increase in isocitrate lyase gene – *icl1* expression and very strict repression of the DevR-DevS regulon suggest that 24 hours postinfection bacteria are still actively dividing and intensively metabolizing in an oxygen-dependent manner, despite activation of some mechanisms sensing increasing CO_2_ levels. The shape of the delineated transcriptome does not resemble the typical changes observed for conditions mimicking persistent macrophage stress (94) such as exposure to nutrient starvation or non-dividing stationary phase, with only mild signs of preparation to withstand low pH and upcoming oxygen depletion. Overall, contrary to previous analyses, our data show that although capable of cometabolizing multiple carbon sources, *M. tuberculosis* at the early stage of infection gives priority to nutrients whose utilization is not as energy-consuming as lipids. Most transcriptomic changes at this stage are oriented into cell surface armor synthesis/remodeling, preventing recognition or intraphagosomal killing.

We have previously shown that *in vitro* opsonization of tubercle bacilli with hSAA-1 affects a moderate set of *Mtb* genes (24). Here, we observed that hSAA-1 opsonization modulates the functional response of *Mtb* to the intracellular environment of macrophages. Among the genes affected by hSAA-1 opsonization during infection, three genes (*rv1195*, *rv2856b*, *rv3093c*) were upregulated exclusively in *Mtb* opsonized with hSAA-1. PE13 encoded by *rv1195* enhances the survival of bacilli under stress conditions such as the presence of H_2_O_2_, SDS, or low pH, and is actively engaged in the interaction between pathogen and host, signaling through the p38-ERK-NF-κB axis, and apoptosis (95). *Rv2856b*/NicT belongs to the family of *Mtb* metal transporters and behaves as a drug efflux pump facilitating cross-resistance to several antibiotics including isoniazid (96) and *Rv3093c* is a SigM-regulated oxidoreductase of unknown function. Among the eight genes (*Rv1405c*, *Rv2661c*, *Rv1684*, *Rv1137c*, *Rv0974c*, *Rv0744ac*, *Rv1815*, *Rv0157a*) exclusively downregulated in hSAA-1 opsonized mycobacteria infecting macrophages, *Rv1405c* encodes a virulence-associated methyltransferase involved in the adaptation of *Mtb* to acid stress (97). *Rv2661c* is involved in phenotypic drug tolerance and associated with *in vivo* infection (98). Among other genes of known function, *Rv1684* is an NO-specific response gene (99). *Rv1137c* may be involved in the posttranslational modification of prenylated proteins (100), *Rv0974c* encodes acyl-CoA carboxylase AccD2, which is probably involved in amino acid biosynthesis (101) and *Rv0744Ac* is a possible transcriptional regulatory protein. Our study also identified 28 genes whose differential expression in response to the intraphagosomal environment was abolished in hSAA-1 opsonized *Mtb* infecting MDMs under the same conditions. Additionally, in this case, most genes represent membrane or secreted antigenic proteins, immunomodulators, and the elements of toxin-antitoxin systems. Interestingly, we found that hSAA-1 opsonization prevents the upregulation of the virulence-related *rv2352c* gene during infection. *Rv2352c* encodes the PPE38 protein that, if overexpressed, inhibits macrophage MHC-I expression and the CD8+ T-cell response (102). This may explain why opsonized but not nonopsonized *Mtb* induces the expression of MHC-I.

Considering our research and literature data, it can be assumed that the observed, elevated SAA-1 level in tuberculosis patients modulates both, the host immune response and the functional response of mycobacteria during infection. The response of macrophages treated with SAA-1 to *Mtb* infection seems to be much stronger and enhanced by the induction of both, innate (MHC-I engagement of natural killer cells) and adaptive (MHC-I through peptides presented to cytotoxic T cells and MHC-II dedicated to adaptive immunity) immune responses (103–105). On the other hand, the opsonization of tubercle bacilli by SAA-1 may facilitate the adaptation of mycobacteria to stress conditions during infection.

## Data availability statement

The original contributions presented in the study are publicly available. This data can be found here: https://www.ncbi.nlm.nih.gov/bioproject/PRJNA1001595.

## Ethics statement

Ethical approval was not required for the studies on humans in accordance with the local legislation and institutional requirements because only commercially available established cell lines were used.

## Author contributions

The concept of the study was designed by BD and JD. Experimental design was performed by MK, RP, BD, and PP. Experiments were performed by MK, RP, PP, BD, JG, KD, and MS. BD, JP, and JD wrote the manuscript. The manuscript was reviewed by all coauthors. All authors contributed to the article and approved the submitted version.

## References

[1] World Health Organization. Global tuberculosis report 2022 (2022). Available at: https://www.who.int/teams/global-tuberculosis-programme/tb-reports/global-tuberculosis-report-2022.

[2] Gengenbacher M Kaufmann SH. Mycobacterium tuberculosis: success through dormancy. FEMS Microbiol Rev (2012) 36(3):514–32. doi: 10.1111/j.1574-6976.2012.00331.x PMC3319523 22320122

[3] World Health Organization. Tuberculosis (2023). Available at: https://www.who.int/news-room/fact-sheets/detail/tuberculosis.

[4] Simper JD Perez E Schlesinger LS Azad AK. Resistance and Susceptibility Immune Factors at Play during Mycobacterium tuberculosis Infection of Macrophages. Pathogens (2022) 11(10):1153. doi: 10.3390/pathogens11101153 36297211 PMC9611686

[5] Moule MG Cirillo JD. Mycobacterium tuberculosis dissemination plays a critical role in pathogenesis. Front Cell Infect Microbiol (2020) 10:65. doi: 10.3389/fcimb.2020.00065 32161724 PMC7053427

[6] Orme IM. A new unifying theory of the pathogenesis of tuberculosis. Tuberculosis (Edinb) (2014) 94(1):8–14. doi: 10.1016/j.tube.2013.07.004 24157189 PMC3877201

[7] Boom WH Schaible UE Achkar JM. The knowns and unknowns of latent Mycobacterium tuberculosis infection. J Clin Invest (2021) 131(3):e136222. doi: 10.1172/JCI136222 33529162 PMC7843221

[8] Singh CR Moulton RA Armitige LY Bidani A Snuggs M Dhandayuthapani S. Processing and presentation of a mycobacterial antigen 85B epitope by murine macrophages is dependent on the phagosomal acquisition of vacuolar proton ATPase and in *situ* activation of cathepsin D. J Immunol (2006) 177(5):3250–9. doi: 10.4049/jimmunol.177.5.3250 16920965

[9] McMurtrey C Harriff MJ Swarbrick GM Duncan A Cansler M Null M. T cell recognition of Mycobacterium tuberculosis peptides presented by HLA-E derived from infected human cells. PloS One (2017) 12(11):e0188288. doi: 10.1371/journal.pone.0188288 29176828 PMC5703486

[10] Leddy O White FM Bryson BD. Immunopeptidomics reveals determinants of Mycobacterium tuberculosis antigen presentation on MHC class I. Elife (2023) 12:e84070. doi: 10.7554/eLife.84070 37073954 PMC10159623

[11] Baena A Porcelli SA. Evasion and subversion of antigen presentation by Mycobacterium tuberculosis. Tissue Antigens (2009) 74(3):189–204. doi: 10.1111/j.1399-0039.2009.01301.x 19563525 PMC2753606

[12] Cooper AM. Cell-mediated immune responses in tuberculosis. Annu Rev Immunol (2009) 27:393–422. doi: 10.1146/annurev.immunol.021908.132703 19302046 PMC4298253

[13] Flynn JL Chan J. Immunology of tuberculosis. Annu Rev Immunol (2001) 19:93–129. doi: 10.1146/annurev.immunol.19.1.93 11244032

[14] Lyadova IV Panteleev AV. Th1 and th17 cells in tuberculosis: protection, pathology, and biomarkers. Mediators Inflamm (2015) 2015:854507. doi: 10.1155/2015/854507 26640327 PMC4657112

[15] Cardona P Cardona PJ. Regulatory T cells in mycobacterium tuberculosis infection. Front Immunol (2019) 10:2139. doi: 10.3389/fimmu.2019.02139 31572365 PMC6749097

[16] De Buck M Gouwy M Wang JM Van Snick J Opdenakker G Struyf S. (SAA) variants and their concentration-dependent functions during host insults. Curr Med Chem (2016) 23(17):1725–55. doi: 10.2174/0929867323666160418114600 PMC5405626 27087246

[17] Eklund KK Niemi K Kovanen PT. Immune functions of serum amyloid A. Crit Rev Immunol (2012) 32(4):335–48. doi: 10.1615/CritRevImmunol.v32.i4.40 23237509

[18] Ganapathi MK Rzewnicki D Samols D Jiang SL Kushner I. Effect of combinations of cytokines and hormones on synthesis of serum amyloid A and C-reactive protein in Hep 3B cells. J Immunol (1991) 147(4):1261–5. doi: 10.4049/jimmunol.147.4.1261 1651357

[19] Migita K Abiru S Nakamura M Komori A Yoshida Y Yokoyama T. Lipopolysaccharide signaling induces serum amyloid A (SAA) synthesis in human hepatocytes *in vitro* . FEBS Lett (2004) 569(1-3):235–9. doi: 10.1016/j.febslet.2004.05.072 15225640

[20] Urieli-Shoval S Linke RP Matzner Y. Expression and function of serum amyloid A, a major acute-phase protein, in normal and disease states. Curr Opin Hematol (2000) 7(1):64–9. doi: 10.1097/00062752-200001000-00012 10608507

[21] Ye RD Sun L. Emerging functions of serum amyloid A in inflammation. J Leukoc Biol (2015) 98(6):923–9. doi: 10.1189/jlb.3VMR0315-080R PMC6608020 26130702

[22] Hari-Dass R Shah C Meyer DJ Raynes JG. Serum amyloid A protein binds to outer membrane protein A of gram-negative bacteria. J Biol Chem (2005) 280(19):18562–7. doi: 10.1074/jbc.M500490200 15705572

[23] Shah C Hari-Dass R Raynes JG. Serum amyloid A is an innate immune opsonin for Gram-negative bacteria. Blood (2006) 108(5):1751–7. doi: 10.1182/blood-2005-11-011932 16735604

[24] Kawka M Brzostek A Dzitko K Kryczka J Bednarek R Plocinska R. Mycobacterium tuberculosis binds human serum amyloid A, and the interaction modulates the colonization of human macrophages and the transcriptional response of the pathogen. Cells (2021) 10(5):1264. doi: 10.3390/cells10051264 34065319 PMC8160739

[25] Korycka-Machala M Viljoen A Pawelczyk J Borowka P Dziadek B Gobis K. 1H-benzo[d]Imidazole derivatives affect mmpL3 in mycobacterium tuberculosis. Antimicrob Agents Chemother (2019) 63(10):e00441-19. 31332069 10.1128/AAC.00441-19 PMC6761528

[26] Pawelczyk J Brzostek A Kremer L Dziadek B Rumijowska-Galewicz A Fiolka M. AccD6, a key carboxyltransferase essential for mycolic acid synthesis in Mycobacterium tuberculosis, is dispensable in a nonpathogenic strain. J Bacteriol (2011) 193(24):6960–72. doi: 10.1128/JB.05638-11 PMC3232849 21984794

[27] Martin M. Cutadapt removes adapter sequences from high-throughput sequencing reads. EMBnet J (2011) 17:10–2. doi: 10.14806/ej.17.1.200

[28] Langmead B Salzberg SL. Fast gapped-read alignment with Bowtie 2. Nat Methods (2012) 9(4):357–9. doi: 10.1038/nmeth.1923 PMC3322381 22388286

[29] Dobin A Davis CA Schlesinger F Drenkow J Zaleski C Jha S. STAR: ultrafast universal RNA-seq aligner. Bioinformatics (2013) 29(1):15–21. doi: 10.1093/bioinformatics/bts635 23104886 PMC3530905

[30] Li H Handsaker B Wysoker A Fennell T Ruan J Homer N. The sequence alignment/map format and SAMtools. Bioinformatics (2009) 25(16):2078–9. doi: 10.1093/bioinformatics/btp352 PMC2723002 19505943

[31] Quinlan AR Hall IM. BEDTools: a flexible suite of utilities for comparing genomic features. Bioinformatics (2010) 26(6):841–2. doi: 10.1093/bioinformatics/btq033 PMC2832824 20110278

[32] Anders S Pyl PT Huber W. HTSeq–a Python framework to work with high-throughput sequencing data. Bioinformatics (2015) 31(2):166–9. doi: 10.1093/bioinformatics/btu638 PMC4287950 25260700

[33] Robinson JT Thorvaldsdottir H Winckler W Guttman M Lander ES Getz G. Integrative genomics viewer. Nat Biotechnol (2011) 29(1):24–6. doi: 10.1038/nbt.1754 PMC3346182 21221095

[34] Powell DR. Degust: interactive RNA-seq analysis. Available at http://degust.erc.monash.edu

[35] Ge SX Son EW Yao R. iDEP: an integrated web application for differential expression and pathway analysis of RNA-Seq data. BMC Bioinf (2018) 19(1):534. doi: 10.1186/s12859-018-2486-6 PMC6299935 30567491

[36] Samaha HMS Elsaid AR Elzehery R Elhelaly R. C-reactive protein and serum amyloid A levels in discriminating Malignant from non-Malignant pleural effusion. Egypt J Chest Dis Tuberc (2015) 64(4):887–92. doi: 10.1016/j.ejcdt.2015.04.004

[37] Marcela Rodriguez G Neyrolles O. Metallobiology of tuberculosis. Microbiol Spectr (2014) 2(3):10.1128/microbiolspec. doi: 10.1128/microbiolspec.MGM2-0012-2013 PMC5180607 26103977

[38] Singh N Sharma N Singh P Pandey M Ilyas M Sisodiya L. HupB, a nucleoid-associated protein, is critical for survival of Mycobacterium tuberculosis under host-mediated stresses and for enhanced tolerance to key first-line antibiotics. Front Microbiol (2022) 13:937970. doi: 10.3389/fmicb.2022.937970 36071978 PMC9441915

[39] Kolodziej M Trojanowski D Bury K Holowka J Matysik W Kakolewska H. Lsr2, a nucleoid-associated protein influencing mycobacterial cell cycle. Sci Rep (2021) 11(1):2910. doi: 10.1038/s41598-021-82295-0 33536448 PMC7858621

[40] Zondervan NA van Dam JCJ Schaap PJ Martins Dos Santos VAP Suarez-Diez M. Regulation of three virulence strategies of mycobacterium tuberculosis: A success story. Int J Mol Sci (2018) 19(2). doi: 10.3390/ijms19020347 PMC5855569 29364195

[41] Vergne I Chua J Lee HH Lucas M Belisle J Deretic V. Mechanism of phagolysosome biogenesis block by viable Mycobacterium tuberculosis. Proc Natl Acad Sci U S A (2005) 102(11):4033–8. doi: 10.1073/pnas.0409716102 PMC554822 15753315

[42] Groschel MI Sayes F Simeone R Majlessi L Brosch R. ESX secretion systems: mycobacterial evolution to counter host immunity. Nat Rev Microbiol (2016) 14(11):677–91. doi: 10.1038/nrmicro.2016.131 27665717

[43] Kurtz S McKinnon KP Runge MS Ting JP Braunstein M. The SecA2 secretion factor of Mycobacterium tuberculosis promotes growth in macrophages and inhibits the host immune response. Infect Immun (2006) 74(12):6855–64. doi: 10.1128/IAI.01022-06 PMC1698048 17030572

[44] Daffe M Crick DC Jackson M. Genetics of capsular polysaccharides and cell envelope (Glyco)lipids. Microbiol Spectr (2014) 2(4):MGM2-0021-2013. doi: 10.1128/9781555818845.ch28 26104202

[45] Hussell T Bell TJ. Alveolar macrophages: plasticity in a tissue-specific context. Nat Rev Immunol (2014) 14(2):81–93. doi: 10.1038/nri3600 24445666

[46] Kawasaki T Ikegawa M Kawai T. Antigen presentation in the lung. Front Immunol (2022) 13:860915. doi: 10.3389/fimmu.2022.860915 35615351 PMC9124800

[47] Hou F Xiao K Tang L Xie L. Diversity of macrophages in lung homeostasis and diseases. Front Immunol (2021) 12:753940. doi: 10.3389/fimmu.2021.753940 34630433 PMC8500393

[48] Mosser DM. The many faces of macrophage activation. J Leukoc Biol (2003) 73(2):209–12. doi: 10.1189/jlb.0602325 12554797

[49] Lambrecht BN. Alveolar macrophage in the driver’s seat. Immunity (2006) 24(4):366–8. doi: 10.1016/j.immuni.2006.03.008 16618595

[50] Rajaram MV Brooks MN Morris JD Torrelles JB Azad AK Schlesinger LS. Mycobacterium tuberculosis activates human macrophage peroxisome proliferator-activated receptor gamma linking mannose receptor recognition to regulation of immune responses. J Immunol (2010) 185(2):929–42. doi: 10.4049/jimmunol.1000866 PMC3014549 20554962

[51] Hoidal JR SD Peterson PK. Phagocytosis, bacterial killing, and metabolism by purified human lung phagocytes. J Infect Diseases (1981) 144:61–71. doi: 10.1093/infdis/144.1.61 7021701

[52] Greening AP LD. Extracellular release of hydrogen peroxide by human alveolar macrophages: the relationship to cigarette smoking and lower respiratory tract infections. Ckin Sci (Lond) (1983) 65:661–4. doi: 10.1042/cs0650661 6627851

[53] Lyons CR BE Toews GB Weissler JC Stastny P Lipscomb MF. Inability of human alveolar macrophages to stimulate resting T cells correlates with decreased antigen-specific T cell-macrophage binding. J Immunol (1986) 137:1173–80. doi: 10.4049/jimmunol.137.4.1173 2426354

[54] Kleinnijenhuis J Oosting M Joosten LA Netea MG Van Crevel R. Innate immune recognition of Mycobacterium tuberculosis. Clin Dev Immunol (2011) 2011:405310. doi: 10.1155/2011/405310 21603213 PMC3095423

[55] Holt PG Oliver J Bilyk N McMenamin C McMenamin PG Kraal G. Downregulation of the antigen presenting cell function(s) of pulmonary dendritic cells *in vivo* by resident alveolar macrophages. J Exp Med (1993) 177(2):397–407. doi: 10.1084/jem.177.2.397 8426110 PMC2190916

[56] Roth MD Golub SH. Human pulmonary macrophages utilize prostaglandins and transforming growth factor beta 1 to suppress lymphocyte activation. J Leukoc Biol (1993) 53(4):366–71. doi: 10.1002/jlb.53.4.366 8482916

[57] Yoshie O Imai T Nomiyama H. Chemokines in immunity. Adv Immunol (2001) 78:57–110. doi: 10.1016/S0065-2776(01)78002-9 11432208

[58] Ansel KM Harris RB Cyster JG. CXCL13 is required for B1 cell homing, natural antibody production, and body cavity immunity. Immunity (2002) 16(1):67–76. doi: 10.1016/S1074-7613(01)00257-6 11825566

[59] LeBlanc HN Ashkenazi A. Apo2L/TRAIL and its death and decoy receptors. Cell Death Differ (2003) 10(1):66–75. doi: 10.1038/sj.cdd.4401187 12655296

[60] Metzemaekers M Vanheule V Janssens R Struyf S Proost P. Overview of the mechanisms that may contribute to the non-redundant activities of interferon-inducible CXC chemokine receptor 3 ligands. Front Immunol (2017) 8:1970. doi: 10.3389/fimmu.2017.01970 29379506 PMC5775283

[61] Mosser DM Zhang X. Interleukin-10: new perspectives on an old cytokine. Immunol Rev (2008) 226:205–18. doi: 10.1111/j.1600-065X.2008.00706.x PMC2724982 19161426

[62] Pu W Zhao C Wazir J Su Z Niu M Song S. Comparative transcriptomic analysis of THP-1-derived macrophages infected with Mycobacterium tuberculosis H37Rv, H37Ra and BCG. J Cell Mol Med (2021) 25(22):10504–20. doi: 10.1111/jcmm.16980 PMC8581329 34632719

[63] Mantovani A Sica A Sozzani S Allavena P Vecchi A Locati M. The chemokine system in diverse forms of macrophage activation and polarization. Trends Immunol (2004) 25(12):677–86. doi: 10.1016/j.it.2004.09.015 15530839

[64] Gordon S Martinez FO. Alternative activation of macrophages: mechanism and functions. Immunity (2010) 32(5):593–604. doi: 10.1016/j.immuni.2010.05.007 20510870

[65] Martinez FO Gordon S. The M1 and M2 paradigm of macrophage activation: time for reassessment. F1000Prime Rep (2014) 6:13. doi: 10.12703/P6-13 24669294 PMC3944738

[66] Lafourcade C Sobo K Kieffer-Jaquinod S Garin J van der Goot FG. Regulation of the V-ATPase along the endocytic pathway occurs through reversible subunit association and membrane localization. PloS One (2008) 3(7):e2758. doi: 10.1371/journal.pone.0002758 18648502 PMC2447177

[67] Zehner M Marschall AL Bos E Schloetel JG Kreer C Fehrenschild D. The translocon protein Sec61 mediates antigen transport from endosomes in the cytosol for cross-presentation to CD8(+) T cells. Immunity (2015) 42(5):850–63. doi: 10.1016/j.immuni.2015.04.008 25979419

[68] Harding CV Boom WH. Regulation of antigen presentation by Mycobacterium tuberculosis: a role for Toll-like receptors. Nat Rev Microbiol (2010) 8(4):296–307. doi: 10.1038/nrmicro2321 20234378 PMC3037727

[69] Walburger A Koul A Ferrari G Nguyen L Prescianotto-Baschong C Huygen K. Protein kinase G from pathogenic mycobacteria promotes survival within macrophages. Science (2004) 304(5678):1800–4. doi: 10.1126/science.1099384 15155913

[70] Sturgill-Koszycki S Schlesinger PH Chakraborty P Haddix PL Collins HL Fok AK. Lack of acidification in Mycobacterium phagosomes produced by exclusion of the vesicular proton-ATPase. Science (1994) 263(5147):678–81. doi: 10.1126/science.8303277 8303277

[71] Schnell DJ Hebert DN. Protein translocons: multifunctional mediators of protein translocation across membranes. Cell (2003) 112(4):491–505. doi: 10.1016/S0092-8674(03)00110-7 12600313

[72] Grotzke JE Harriff MJ Siler AC Nolt D Delepine J Lewinsohn DA. The Mycobacterium tuberculosis phagosome is a HLA-I processing competent organelle. PloS Pathog (2009) 5(4):e1000374. doi: 10.1371/journal.ppat.1000374 19360129 PMC2661020

[73] Harriff MJ Burgdorf S Kurts C Wiertz EJ Lewinsohn DA Lewinsohn DM. TAP mediates import of Mycobacterium tuberculosis-derived peptides into phagosomes and facilitates loading onto HLA-I. PloS One (2013) 8(11):e79571. doi: 10.1371/journal.pone.0079571 24244525 PMC3823705

[74] Ravesloot-Chavez MM Van Dis E Stanley SA. The innate immune response to mycobacterium tuberculosis infection. Annu Rev Immunol (2021) 39:611–37. doi: 10.1146/annurev-immunol-093019-010426 33637017

[75] Reiling N Holscher C Fehrenbach A Kroger S Kirschning CJ Goyert S. Cutting edge: Toll-like receptor (TLR)2- and TLR4-mediated pathogen recognition in resistance to airborne infection with Mycobacterium tuberculosis. J Immunol (2002) 169(7):3480–4. doi: 10.4049/jimmunol.169.7.3480 12244136

[76] Sugawara I Yamada H Li C Mizuno S Takeuchi O Akira S. Mycobacterial infection in TLR2 and TLR6 knockout mice. Microbiol Immunol (2003) 47(5):327–36. doi: 10.1111/j.1348-0421.2003.tb03404.x 12825894

[77] Feng CG Scanga CA Collazo-Custodio CM Cheever AW Hieny S Caspar P. Mice lacking myeloid differentiation factor 88 display profound defects in host resistance and immune responses to Mycobacterium avium infection not exhibited by Toll-like receptor 2 (TLR2)- and TLR4-deficient animals. J Immunol (2003) 171(9):4758–64. doi: 10.4049/jimmunol.171.9.4758 14568952

[78] Bafica A Scanga CA Feng CG Leifer C Cheever A Sher A. TLR9 regulates Th1 responses and cooperates with TLR2 in mediating optimal resistance to Mycobacterium tuberculosis. J Exp Med (2005) 202(12):1715–24. doi: 10.1084/jem.20051782 PMC2212963 16365150

[79] Baumann CL Aspalter IM Sharif O Pichlmair A Bluml S Grebien F. CD14 is a coreceptor of Toll-like receptors 7 and 9. J Exp Med (2010) 207(12):2689–701. doi: 10.1084/jem.20101111 PMC2989773 21078886

[80] Maeda N Nigou J Herrmann JL Jackson M Amara A Lagrange PH. The cell surface receptor DC-SIGN discriminates between Mycobacterium species through selective recognition of the mannose caps on lipoarabinOmannan. J Biol Chem (2003) 278(8):5513–6. doi: 10.1074/jbc.C200586200 12496255

[81] Kang PB Azad AK Torrelles JB Kaufman TM Beharka A Tibesar E. The human macrophage mannose receptor directs Mycobacterium tuberculosis lipoarabinOmannan-mediated phagosome biogenesis. J Exp Med (2005) 202(7):987–99. doi: 10.1084/jem.20051239 PMC2213176 16203868

[82] Doz E Rose S Nigou J Gilleron M Puzo G Erard F. Acylation determines the toll-like receptor (TLR)-dependent positive versus TLR2-, mannose receptor-, and SIGNR1-independent negative regulation of pro-inflammatory cytokines by mycobacterial lipOmannan. J Biol Chem (2007) 282(36):26014–25. doi: 10.1074/jbc.M702690200 17617634

[83] Yonekawa A Saijo S Hoshino Y Miyake Y Ishikawa E Suzukawa M. Dectin-2 is a direct receptor for mannose-capped lipoarabinOmannan of mycobacteria. Immunity (2014) 41(3):402–13. doi: 10.1016/j.immuni.2014.08.005 25176311

[84] Twigg MW Freestone K Homer-Vanniasinkam S Ponnambalam S. The LOX-1 scavenger receptor and its implications in the treatment of vascular disease. Cardiol Res Pract (2012) 2012:632408. doi: 10.1155/2012/632408 22454776 PMC3290926

[85] Palanisamy GS Kirk NM Ackart DF Obregon-Henao A Shanley CA Orme IM. Uptake and accumulation of oxidized low-density lipoprotein during Mycobacterium tuberculosis infection in Guinea pigs. PloS One (2012) 7(3):e34148. doi: 10.1371/journal.pone.0034148 22493658 PMC3320102

[86] Marcais A Coupet CA Walzer T Tomkowiak M Ghittoni R Marvel J. Cell-autonomous CCL5 transcription by memory CD8 T cells is regulated by IL-4. J Immunol (2006) 177(7):4451–7. doi: 10.4049/jimmunol.177.7.4451 16982880

[87] Appay V Rowland-Jones SL. RANTES: a versatile and controversial chemokine. Trends Immunol (2001) 22(2):83–7. doi: 10.1016/S1471-4906(00)01812-3 11286708

[88] Du P Sohaskey CD Shi L. Transcriptional and Physiological Changes during Mycobacterium tuberculosis Reactivation from Non-replicating Persistence. Front Microbiol (2016) 7:1346. doi: 10.3389/fmicb.2016.01346 27630619 PMC5005354

[89] Minnikin DE Kremer L Dover LG Besra GS. The methyl-branched fortifications of Mycobacterium tuberculosis. Chem Biol (2002) 9(5):545–53. doi: 10.1016/S1074-5521(02)00142-4 12031661

[90] Zimmermann M Kogadeeva M Gengenbacher M McEwen G Mollenkopf HJ Zamboni N. Integration of Metabolomics and Transcriptomics Reveals a Complex Diet of Mycobacterium tuberculosis during Early Macrophage Infection. mSystems (2017) 2(4):e00057-17. doi: 10.1128/mSystems.00057-17 28845460 PMC5566787

[91] Lopez-Agudelo VA Baena A Barrera V Cabarcas F Alzate JF Beste DJV. Dual RNA sequencing of mycobacterium tuberculosis-infected human splenic macrophages reveals a strain-dependent host-pathogen response to infection. Int J Mol Sci (2022) 23(3):1803. doi: 10.3390/ijms23031803 35163725 PMC8836425

[92] Rienksma RA Suarez-Diez M Mollenkopf HJ Dolganov GM Dorhoi A Schoolnik GK. Comprehensive insights into transcriptional adaptation of intracellular mycobacteria by microbe-enriched dual RNA sequencing. BMC Genomics (2015) 16(1):34. doi: 10.1186/s12864-014-1197-2 25649146 PMC4334782

[93] Medley J Goff A Bettencourt PJG Dare M Cole L Cantillon D. Dissecting the mycobacterium bovis BCG response to macrophage infection to help prioritize targets for anti-tuberculosis drug and vaccine discovery. Vaccines (Basel) (2022) 10(1):113. doi: 10.3390/vaccines10010113 35062774 PMC8780277

[94] Vilcheze C Yan B Casey R Hingley-Wilson S Ettwiller L Jacobs WR Jr. Commonalities of mycobacterium tuberculosis transcriptomes in response to defined persisting macrophage stresses. Front Immunol (2022) 13:909904. doi: 10.3389/fimmu.2022.909904 35844560 PMC9283954

[95] Li H Li Q Yu Z Zhou M Xie J. Mycobacterium tuberculosis PE13 (Rv1195) manipulates the host cell fate via p38-ERK-NF-kappaB axis and apoptosis. Apoptosis (2016) 21(7):795–808. doi: 10.1007/s10495-016-1249-y 27147522

[96] Adhikary A Biswal S Chatterjee D Ghosh AS. A NiCoT family metal transporter of Mycobacterium tuberculosis (Rv2856/NicT) behaves as a drug efflux pump that facilitates cross-resistance to antibiotics. Microbiol (Reading) (2022) 168(10). doi: 10.1099/mic.0.001260 36282241

[97] Healy C Golby P MacHugh DE Gordon SV. The MarR family transcription factor Rv1404 coordinates adaptation of Mycobacterium tuberculosis to acid stress via controlled expression of Rv1405c, a virulence-associated methyltransferase. Tuberculosis (Edinb) (2016) 97:154–62. doi: 10.1016/j.tube.2015.10.003 26615221

[98] Pisu D Huang L Narang V Theriault M Le-Bury G Lee B. Single cell analysis of M. tuberculosis phenotype and macrophage lineages in the infected lung. J Exp Med (2021) 218(9):e20210615. 34292313 10.1084/jem.20210615 PMC8302446

[99] Voskuil MI Bartek IL Visconti K Schoolnik GK. The response of mycobacterium tuberculosis to reactive oxygen and nitrogen species. Front Microbiol (2011) 2:105. doi: 10.3389/fmicb.2011.00105 21734908 PMC3119406

[100] Manganelli R. Sigma factors: key molecules in mycobacterium tuberculosis physiology and virulence. Microbiol Spectr (2014) 2(1):MGM2–0007-2013. doi: 10.1128/9781555818845.ch7 26082107

[101] Ehebauer MT Zimmermann M Jakobi AJ Noens EE Laubitz D Cichocki B. Characterization of the mycobacterial acyl-CoA carboxylase holo complexes reveals their functional expansion into amino acid catabolism. PloS Pathog (2015) 11(2):e1004623. doi: 10.1371/journal.ppat.1004623 25695631 PMC4347857

[102] Meng L Tong J Wang H Tao C Wang Q Niu C. PPE38 protein of mycobacterium tuberculosis inhibits macrophage MHC class I expression and dampens CD8(+) T cell responses. Front Cell Infect Microbiol (2017) 7:68. doi: 10.3389/fcimb.2017.00068 28348981 PMC5346565

[103] Jiang J Natarajan K Margulies DH. MHC molecules, T cell receptors, natural killer cell receptors, and viral immunoevasins-key elements of adaptive and innate immunity. Adv Exp Med Biol (2019) 1172:21–62. doi: 10.1007/978-981-13-9367-9_2 31628650

[104] Uzhachenko RV Shanker A. CD8(+) T lymphocyte and NK cell network: circuitry in the cytotoxic domain of immunity. Front Immunol (2019) 10:1906. doi: 10.3389/fimmu.2019.01906 31456803 PMC6700470

[105] Leddon SA Sant AJ. Generation of MHC class II-peptide ligands for CD4 T-cell allorecognition of MHC class II molecules. Curr Opin Organ Transplant (2010) 15(4):505–11. doi: 10.1097/MOT.0b013e32833bfc5c PMC3085167 20616724

