# Supplementary material for: The functional response of human monocyte-derived macrophages to serum amyloid A and Mycobacterium tuberculosis infection
Source: Front Immunol. 2023 Sep 15;14:1238132. doi: 10.3389/fimmu.2023.1238132 (PMC10540855; doi:10.3389/fimmu.2023.1238132)
Supplement: Supplementary file 1 [file DataSheet_1.pdf]

Supplementary Material

The functional response of human monocyte-derived macrophages to serum amyloid A and infection with *Mycobacterium tuberculosis*.

Malwina Kawka, Renata Płocińska, Przemysław Płociński, Jakub Pawełczyk, Marcin Słomka3, Justyna Gatkowska, Katarzyna Dzitko, Bożena Dziadek* and Jarosław Dziadek *
Correspondence: Corresponding Author: bozena.dziadek@biol.uni.lodz.pl ; jdziadek@cbm.pan.pl

1 Supplementary Data

Figure S1. Principal component analysis (PCA) of the repeats of uninfected MDMs (left panel) and MDMs infected with Mtb (right panel).
Figure S2. Principal component analysis (PCA) of all samples.
Figure S3. Global analysis of differentially expressed genes in MDMs infected with Mtb, infected with Mtb opsonized by hSAA-1 or treated with hSAA-1.
Figure S4 Cytokines and cytokine receptors of Mtb infected MDMs.
Figure S5 Cytokines and cytokine receptors of MDMs treated with hSAA-1.
Figure S6 Cytokines and cytokine receptors of MDMs infected with hSAA-1 opsonized Mtb.
Figure S7. The concentrations of selected cytokines were determined using the Milliplex system.
Figure S8 Global analysis of total RNA isolated from tubercle bacilli.
Table 1 Gene list and corresponding TaqMan probe catalog numbers for RTPCR analysis.
Table S2 Differentially expressed gene (DEG) analysis between individual donors.
Table S3 – separate dataset, Excel file
Table S4 Differentially expressed genes in *Mtb* upon infection.
Table S5 -The opsonization of *Mtb* with hSAA-1 enhances the response of MDMs postinfection.

Supplementary Figures and Tables

1.1 Supplementary Figures

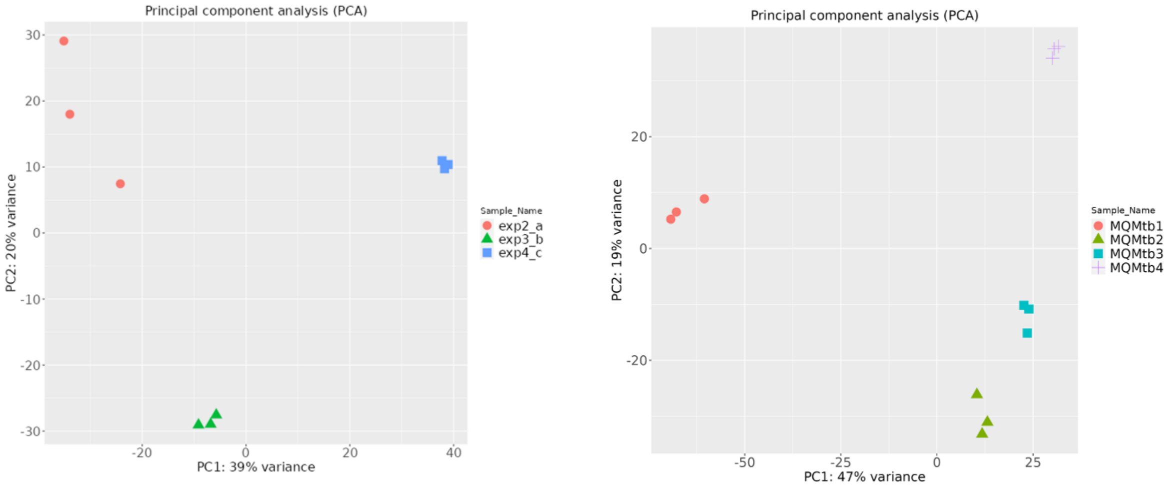

Supplementary Figure 1. Principal component analysis (PCA) of the repeats of uninfected MDMs (left panel) and MDMs infected with Mtb (right panel). Each repeat of the individual donor is marked by orange circle, green triangle, blue square, and purple cross. The analysis was completed using iDEP.96 platform.

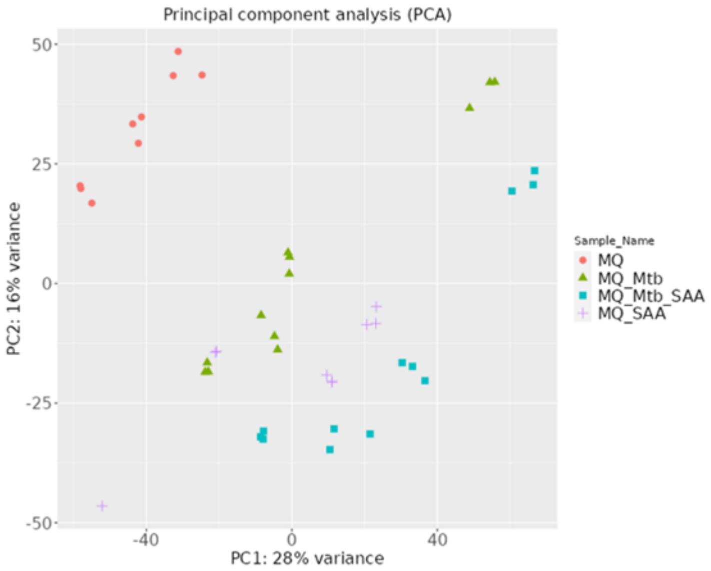

Supplementary Figure 2. Principal component analysis (PCA) of all samples. Each repeat of uninfected MDMs (MQ) is marked by orange circle, MDMs infected with Mtb (MQ_Mtb) by green triangle, MDMs infected with Mtb opsonized with hSAA-1 (MQ_Mtb_SAA) by blue square, and MDMs treated with hSAA-1 (MQ_SAA) by purple cross. The analysis was completed using iDEP.96 platform.

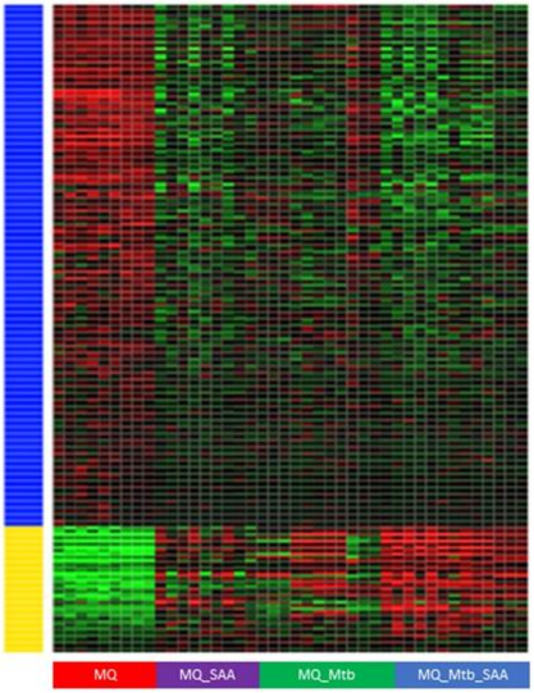

Supplementary Figure 3. The global analysis of differential expressed genes in MDMs infected with Mtb, infected with Mtb opsonized by hSAA-1 or treated with hSAA-1. The up (429) and down (1392) regulated genes are marked by yellow and blue rectangle, respectively. MQ - uninfected MDMs, MQ_Mtb - MDMs infected with Mtb, MQ_SAA - MDMs treated with hSAA-1, and MQ_Mtb_SAA MDMs infected with Mtb opsonized with hSAA-1. The analysis was completed using iDEP.96 platform.

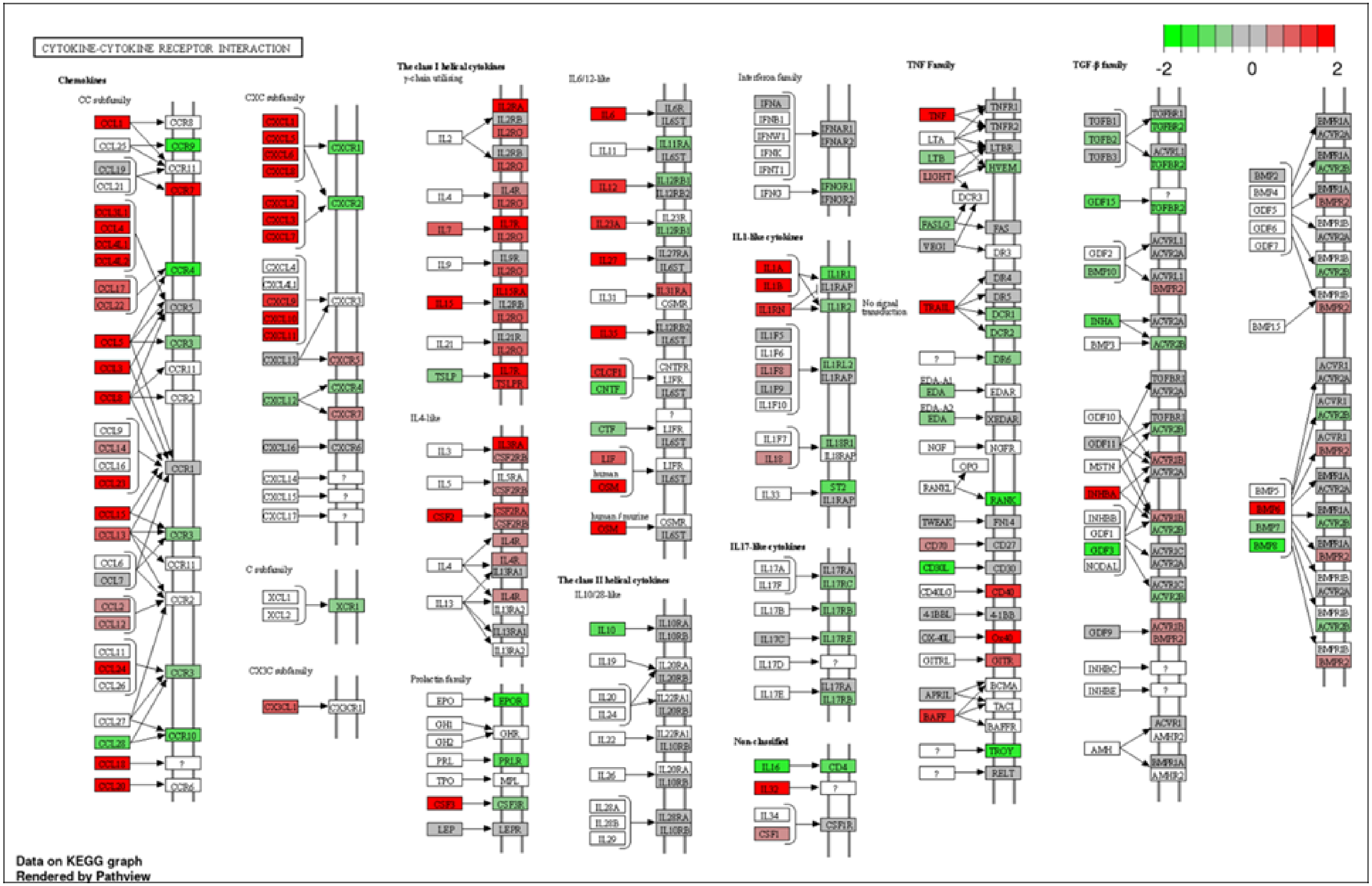

Supplementary Figure 4. Cytokine and cytokine receptors of MDMs infected with *Mtb*. The analysis was completed using iDEP.96 platform based on total RNA sequencing isolated from MDMs of three (control MDMs) or four (Mtb infected MDMs) blood donors in three biological repeats each.

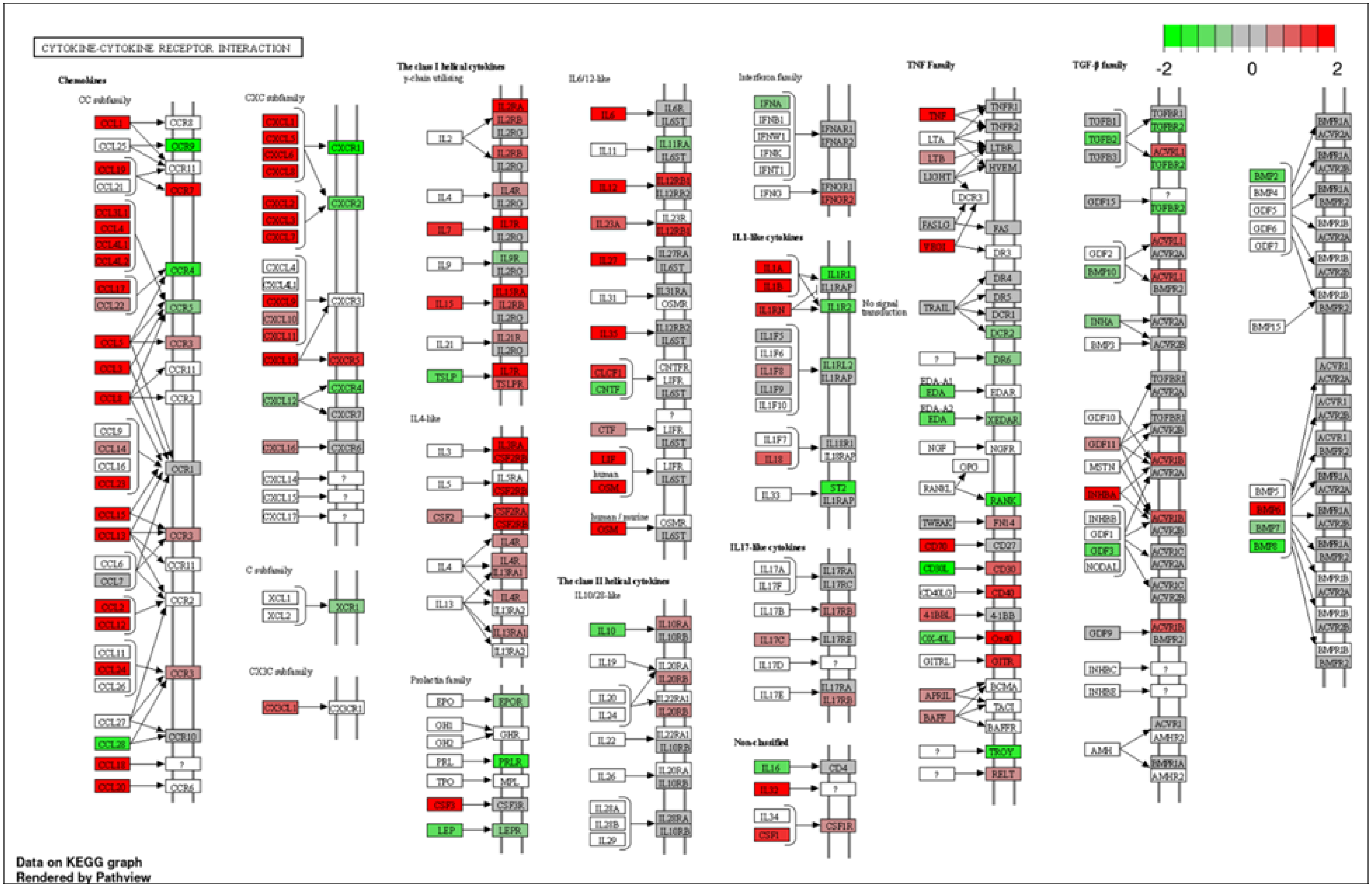

Supplementary Figure 5. Cytokine and cytokine receptors of hSAA-1 treated MDMs. The gene expression profile of MDMs treated with hSAA-1 was compared to control MDMs. The analysis was completed using iDEP.96 platform based on total RNA sequencing of MDMs of three blood donors in three biological repeats each.

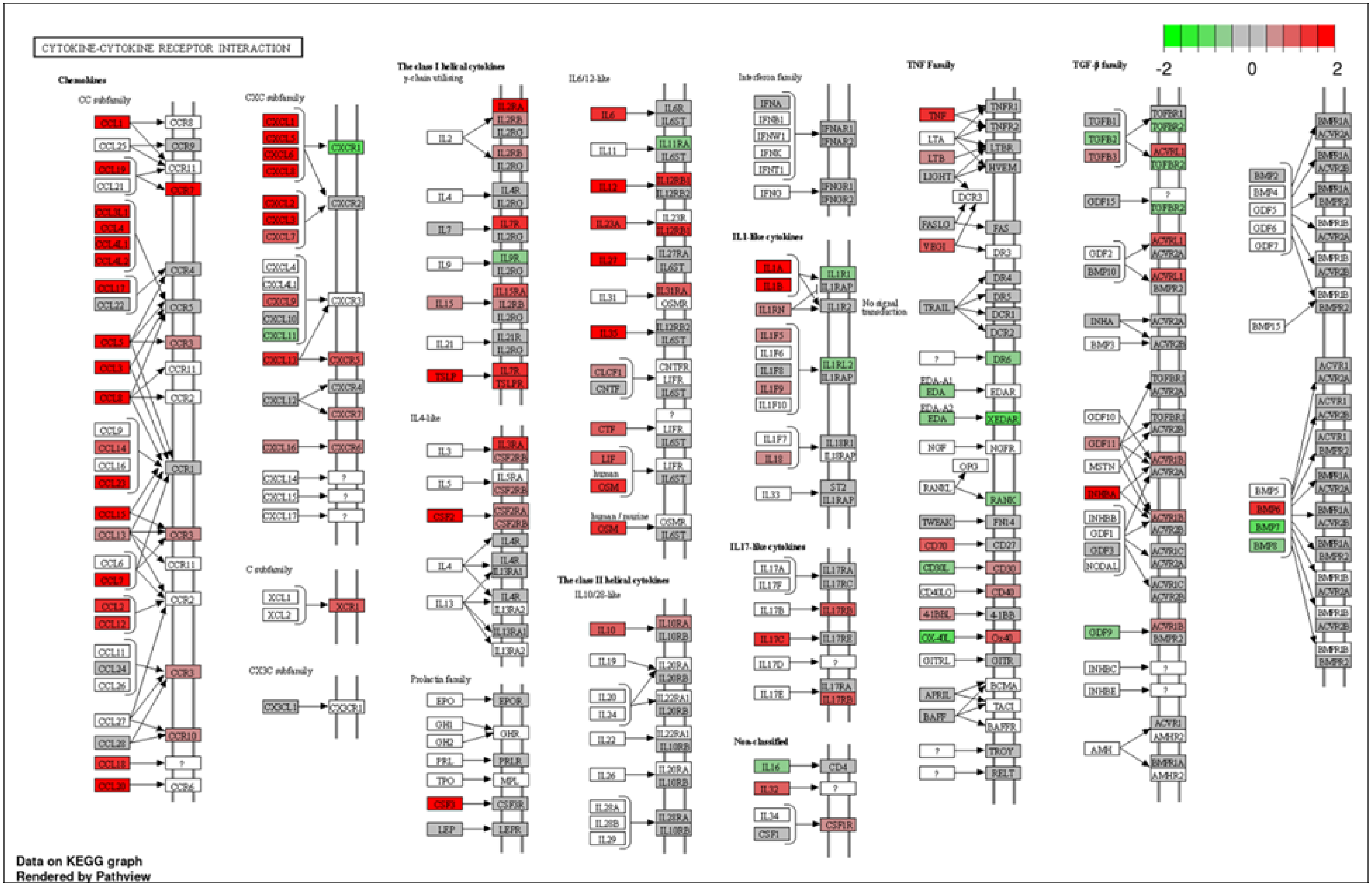

Supplementary Figure 6. Cytokine and cytokine receptors of MDMs infected with hSAA-1 opsonized Mtb. The gene expression of MDMs infected with hSAA-1 opsonized Mt was compared to the MDMs infected with non-opsonized Mtb. The analysis was completed using iDEP.96 platform based on total RNA sequencing isolated from MDMs of four blood donors completed in three biological repeats each.

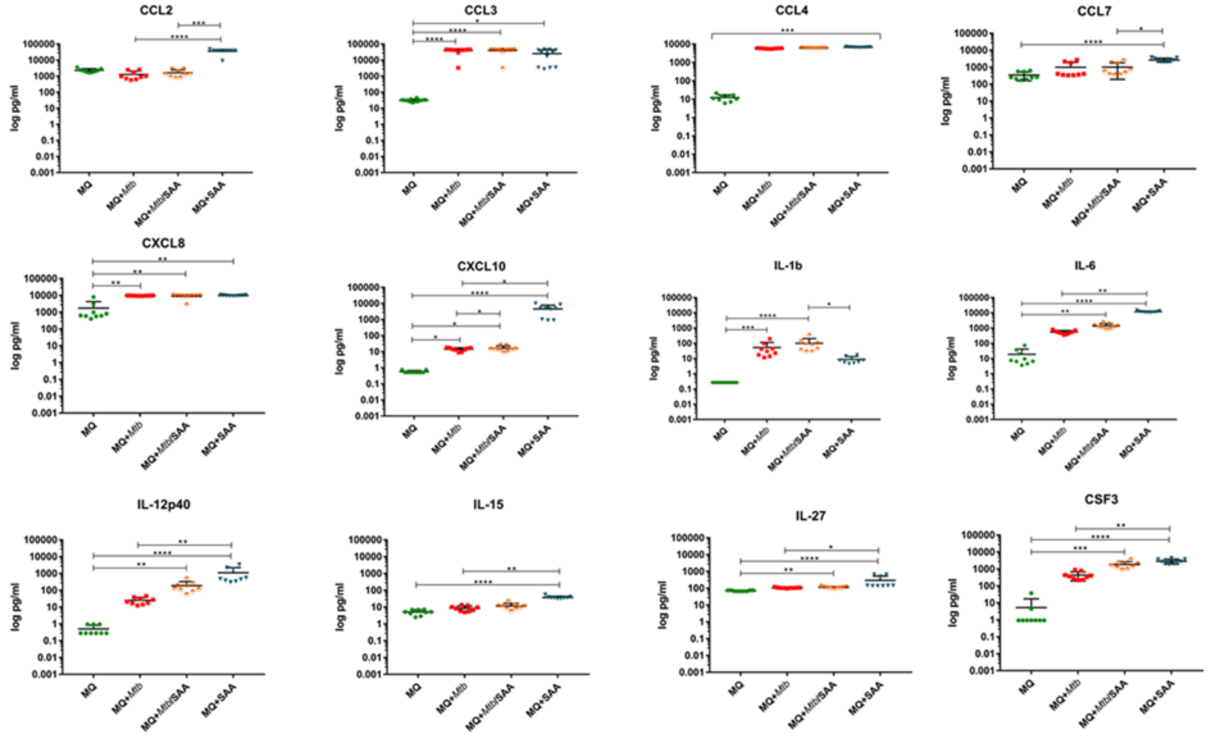

Supplementary Figure 7. The concentrations of selected cytokines determined using Milliplex system. The protein level was assessed in the cell supernatant of control MDMs (MQ), MDMs infected with non-opsonized Mtb (MQ+Mtb), hSAA-1 opsonized Mtb (MQ+Mtb/SAA), and MDMs treated with hSAA-1 (MQ+SAA). The assay was performed for three independent healthy blood donors and the samples of collected culture supernatants were run in triplicate. The data distribution was evaluated by Shapiro-Wilk normality test. Further, statistical analysis was performed by Kruskal-Wallis one-way ANOVA with post-hoc Dunn's test or one-way ANOVA with post-hoc Tukey test (CCL4). *represents p<0.05, **p<0.0021, ***p<0.0002, ****p<0.0001, respectively.

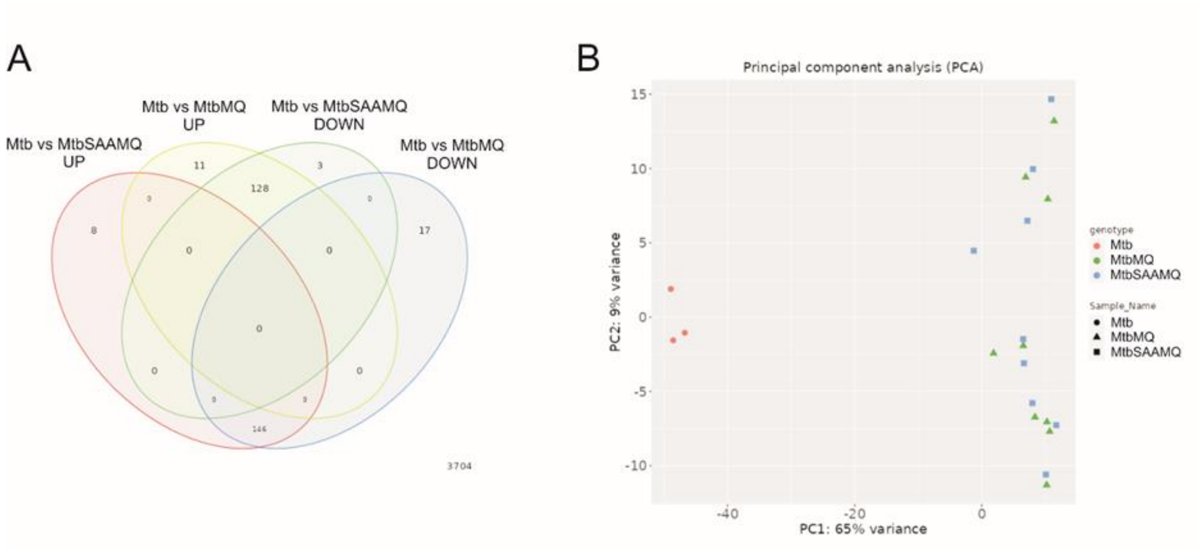

Supplementary Figure 8. Global analysis of total RNAs isolated from tubercle bacilli. (A) Venn diagram presenting differential gene expression of control Mtb compared to non-opsonized and hSAA-1 opsonized Mtb isolated from MDMs (MtbMQ and MtbSAAMQ, respectively). (B) Principal component analysis (PCA) of the triplicates of control Mtb, Mtb and hSAA-1 opsonized Mtb isolated from MDMs of three individual blood donors. Each triplicate is marked by orange circle (Mtb), green triangle (MtbMQ), blue square (MtbSAAMQ). The analysis was completed using iDEP.96 platform.

1.2 Supplementary Tables

Supplementary Table 1. Gene list and corresponding Taqman probe catalogue numbers for RT-PCR analysis.

Gene	Name	Tagman assay catalog number
CXCL8	Interleukin-8	Hs00174103_m1
CCL19	Chemokine ligand 19	Hs00171149_m1
CSF2	Colony Stimulating Factor 2	Hs00929873_m1
GAPDH	Glyceraldehyde 3-phosphate dehydrogenase	Hs02786624_g1

Supplementary Table 2. Differential expressed genes (DEGs) analysis between individual donors.

**A-Comparisons**	**Up**	**Down**
MQMtb4-MQMtb3	148	389
MQMtb2-MQMtb3	184	129
MQMtb1-MQMtb3	232	1004
MQMtb4-MQMtb2	201	613
MQMtb3-MQMtb4	389	148
MQMtb3-MQMtb2	129	184
MQMtb1-MQMtb4	356	936
MQMtb1-MQMtb2	156	1062
MQ3-MQ4	446	200
MQ2-MQ4	1980	149
MQ2-MQ3	476	73

MQMtb represents MDMs infected with *Mtb*. MQ represents uninfected MDMs.

The analysis was completed using iDEP.96 platform.

Supplementary Table 3. Data file – separate excel file

Supplementary Table 4. Differently expressed genes in *Mtb* upon infection.

A - hSAA-1 opsonization-induced differentially expressed genes in bacilli upon infection

Up-regulated genes			Down-regulated genes		
**number**	**name**	**protein function**	**number**	**name**	**protein function**
Rv1195	*pe13*		Rv1405C		MarR family transcription factor
Rv3093C		oxidoreductase	Rv1815		secreted cell wall protein
Rv2856B	*nicT*	NiCoT family metal transporter	Rv0157A		
			Rv0974C	*accD2*	
			Rv2661C		
			Rv1684		NO-Specific Response gene
			Rv1137C		
			Rv0744AC		Possible transcriptional regulatory protein

B - genes that are differentially expressed only in non-opsonized bacilli upon infection

Rv1778C			Rv3868	*eccA1*	ATPase from the ESX-1 secretion system
Rv2258C		S-adenosyl-l-methionine-dependent methyltransferase	Rv1863C		membrane protein
Rv3823C			Rv3683		membrane protein
Rv3310	*sapM*		Rv2575		membrane protein
Rv3316	*sdhC*	succinate dehydrogenase/fumarate reductase complex	Rv1159	*pimE*	membrane protein, mannosyltransferase
Rv0156	*pntAb*	pyridine nucleotide transhydrogenase subunit alpha	Rv3700C		
Rv1114	*vapC32*	Ribonuclease, Vap toxin-anti-toxin system	Rv1620C	*cydC*	Transmembrane, ATP-binding ABC transporter

Rv1115		antigenic membrane protein	Rv2827C		
Rv2352C	*ppe38*		Rv1062		
Rv2058C	*rpmB2*	50S ribosomal protein	Rv2601A	*vapB41*	Vap toxin-anti-toxin system
Rv3187		probable transposase	Rv0815C	*cysA2*	Secreted protein, Putative thiosulfate:cyanide sulfurtransferase
			Rv1040C	*ppe8*	
			Rv0329C		
			Rv2401A		membrane protein
			Rv1952	*vapB14*	Vap toxin-anti-toxin system
			Rv2811		
			Rv1587C		

Supplementary Table 5. The opsonization of *Mtb* with hSAA-1 enhance the response of MDMs postinfection.

		MQ vs MQ+Mtb	MQ vs MQ+SAA	MQMtb vs MQ+MtbSAA
**TNF**	tumor necrosis factor	3,53	2,47	1,37
**CCL5**	C-C motif chemokine ligand 5	5,05	6,92	2,03
**CXCL8**	C-X-C motif chemokine ligand 8	5,66	6,73	2,39
**CCL4**	C-C motif chemokine ligand 4	3,93	4,6	1,39
**CSF2/GMCSF**	colony stimulating factor 2	4	0,79	1,71
**IL15**	interleukin 15	2,04	1,67	1,29
**IL27**	interleukin 27	2,68	0,72	1,43
**IL1B**	interleukin 1 beta	5,03	7,67	2,77
**CXCL10**	C-X-C motif chemokine ligand 10	2,97	0,62	1,08
**IL6**	interleukin 6	4,42	4,72	1,32
**CSF3/GCSF**	colony stimulating factor 3	3,65	3,17	2,47
**IL1A**	interleukin 1 alpha	2,07	3,24	1,68
**CCL3**	C-C motif chemokine ligand 3	1,39	2,25	1,98
**IL12B**	interleukin 12B	1,55	2,71	4,22
**CCL2**	C-C motif chemokine ligand 2	0,74	2,07	3,09
**CCL7**	C-C motif chemokine ligand 7	-0,01	0,4	1,64

Heat map based on transcriptomic analysis of MDMs infected with non-opsonized *Mtb* compared to control MDMs (MQ vs MQ+Mtb), MDMs treated with hSAA-1 compared to control MDMs (MQ vs MQ+SAA), opsonized Mtb (MQ+Mtb/SAA), and MDMs infected with hSAA-1-opsonized *Mtb* compared to MDMs infected with non-opsonized (MQMtb vs MQ+MtbSAA). Total RNA was isolated from MDMs of three (control MDMs, MDMs treated with hSAA-1) or four (non-opsonized and hSAA-1 opsonized Mtb infected MDMs) blood donors in three biological repeats each.
